# Efficacy of Telephone-Based Cognitive Behavioral Therapy for Weight Loss, Disordered Eating, and Psychological Distress After Bariatric Surgery: A Randomized Clinical Trial

**DOI:** 10.1001/jamanetworkopen.2023.27099

**Published:** 2023-08-03

**Authors:** Sanjeev Sockalingam, Samantha E. Leung, Clement Ma, George Tomlinson, Raed Hawa, Susan Wnuk, Timothy Jackson, David Urbach, Allan Okrainec, Jennifer Brown, Daniella Sandre, Stephanie E. Cassin

**Affiliations:** 1 Bariatric Surgery Program, University Health Network, Toronto, Ontario, Canada; 2 Department of Psychiatry, University of Toronto, Toronto, Ontario, Canada; 3 Centre for Addiction and Mental Health, Toronto, Ontario, Canada; 4 Centre for Mental Health, University Health Network, Toronto, Ontario, Canada; 5 Division of Biostatistics, Dalla Lana School of Public Health, University of Toronto, Toronto, Ontario, Canada; 6 Institute of Health Policy, Management and Evaluation, Dalla Lana School of Public Health, University of Toronto, Toronto, Ontario, Canada; 7 Division of General Surgery, University Health Network, University of Toronto, Toronto, Ontario, Canada; 8 Department of Surgery, Women’s College Hospital, Toronto, Canada; 9 Ottawa Hospital Bariatric Centre of Excellence, Ottawa, Ontario, Canada; 10 Department of Psychology, Toronto Metropolitan University, Toronto, Ontario, Canada

## Abstract

**Importance:**

Weight regain after bariatric surgery is associated with recurrence of obesity-related medical comorbidities and deterioration in quality of life. Developing efficacious psychosocial interventions that target risk factors, prevent weight regain, and improve mental health is imperative.

**Objective:**

To determine the efficacy of a telephone-based cognitive behavioral therapy (tele-CBT) intervention at 1 year after bariatric surgery in improving weight loss, disordered eating, and psychological distress.

**Design, Setting, and Participants:**

This multisite randomized clinical trial was conducted at 3 hospital-based bariatric surgery programs, with recruitment between February 2018 and December 2021. Eligibility for participation was assessed among 314 adults at 1 year after bariatric surgery who were fluent in English and had access to a telephone and the internet. Patients with active suicidal ideation or poorly controlled severe psychiatric illness were excluded. Primary and secondary outcome measures were assessed at baseline (1 year after surgery), after the intervention (approximately 15 months after surgery), and at 3-month follow-up (approximately 18 months after surgery). Data were analyzed from January to February 2023.

**Interventions:**

The tele-CBT intervention consisted of 6 weekly 1-hour sessions and a seventh booster session 1 month later. The control group received standard postoperative bariatric care.

**Main Outcomes and Measures:**

The primary outcome was postoperative percentage total weight loss. Secondary outcomes were disordered eating (Binge Eating Scale [BES] and Emotional Eating Scale [EES]) and psychological distress (Patient Health Questionnaire-9 item scale [PHQ-9] and Generalized Anxiety Disorder-7 item scale [GAD-7]). The hypotheses and data-analytic plan were developed prior to data collection.

**Results:**

Among 306 patients 1 year after bariatric surgery (255 females [83.3%]; mean [SD] age, 47.55 [9.98] years), there were 152 patients in the tele-CBT group and 154 patients in the control group. The group by time interaction for percentage total weight loss was not significant (*F*_1,160.61_ = 2.09; *P* = .15). However, there were significant interactions for mean BES (*F*_2,527.32_ = 18.73; *P* < .001), EES total (*F*_2,530.67_ = 10.83; *P* < .001), PHQ-9 (*F*_2,529.93_ = 17.74; *P* < .001), and GAD-7 (*F*_2,535.16_ = 15.29; *P* < .001) scores between the tele-CBT group and control group across all times.

**Conclusions and Relevance:**

This study found that tele-CBT delivered at 1 year after surgery resulted in no change in short-term weight outcomes but improved disordered eating and psychological distress. The impact of these psychosocial improvements on longer-term weight outcomes is currently being examined as part of this longitudinal multisite randomized clinical trial.

**Trial Registration:**

ClinicalTrials.gov Identifier: NCT03315247

## Key Points

**Question:**

How effective is a telephone-based cognitive behavioral therapy (tele-CBT) intervention delivered at 1 year after bariatric surgery, in improving weight loss, disordered eating, and psychological distress?

**Findings:**

In this randomized clinical trial that included 306 adults 1 year after bariatric surgery, weight outcomes were not significantly different between groups. However, binge eating, emotional eating, and depression and anxiety symptoms were significantly reduced in individuals receiving tele-CBT vs the control group.

**Meaning:**

This study found that tele-CBT delivered 1 year after surgery resulted in no change in short-term weight outcomes but improved disordered eating and psychological distress.

## Teaser

This randomized clinical trial investigates the efficacy of a telephone-based cognitive therapy intervention in improving weight loss among patients who underwent bariatric surgery.

## Introduction

Obesity is a significant global health concern.^1^ Despite advances in pharmacotherapy and behavioral treatments for obesity and related comorbidities, bariatric surgery remains the most effective treatment for severe obesity.^2,3^ In addition to reducing metabolic disease, it reduces mortality in patients with severe obesity.^4,5^ However, 11% to 22% of patients experienced suboptimal weight loss within the first 2 years after surgery, and 10% or greater weight regain was reported by 23% and 72% of patients at 1 year and 5 years after surgery, respectively.^6^

Weight regain is a major challenge facing bariatric teams. A systematic review identified 5 categories of risk factors associated with weight regain: temporal, anatomical, genetic, dietary, and psychiatric factors.^7^ Binge eating, loss-of-control eating, emotional eating, food urges, larger portion sizes, and anxiety were the modifiable dietary and psychiatric risk factors most consistently associated with weight regain.^7^ Furthermore, an additional review suggested that the most common factors associated with weight regain were dysregulated or maladaptive eating behaviors, lifestyle factors, life stresses, and depressive symptoms.^6^ Data from systematic reviews and meta-analyses concluded that post–bariatric surgery eating psychopathology was associated with weight regain^8^ and showed an association between depression and disordered eating, lower mental quality of life, and weight regain.^9^

Given that weight regain has been associated with recurrence of medical comorbidities and deterioration in quality of life,^6,10,11^ developing efficacious psychosocial interventions that target risk factors and prevent or reverse weight regain is imperative.^6^ Several studies have examined the effectiveness of psychosocial interventions for improving disordered eating and psychological distress before and after bariatric surgery. Concerning timing of psychosocial interventions, those delivered before bariatric surgery did not yield sustained benefits in improved disordered eating, psychological distress, or weight loss after bariatric surgery compared with surgery alone.^7,8^ Psychosocial interventions delivered 1 year after surgery were likely associated with improved disordered eating and psychological distress^12,13^ and potentially mitigation of significant weight regain, although the current evidence for weight loss outcomes is mixed.^13^ A systematic review that included randomized clinical trials (RCTs) examining the efficacy of psychosocial interventions for disordered eating in adult patients who underwent bariatric surgery identified a total of 7 studies (2 preoperative and 5 postoperative studies), with the largest study sample consisting of 117 patients.^14^ Findings from this systematic review included support for short-term reductions in eating pathology before and after surgery; however, it was noted that there were a small number of RCTs and that additional longitudinal studies are needed.^14^ Moreover, only 2 of the postsurgery psychosocial interventions used web-based modules, telephone communication, or videoconferencing, which has expanded during the COVID-19 pandemic.^14^ Larger, multisite RCTs that are exclusively virtual have not been published to date, to our knowledge.

This multisite RCT sought to determine the efficacy of a telephone-based cognitive behavioral therapy (tele-CBT) intervention delivered 1 year after bariatric surgery in improving weight loss, disordered eating (ie, binge eating and emotional eating), and psychological distress (ie, depressive and anxiety symptoms) at 1.5 years after surgery compared with a treatment-as-usual control group (ie, standard postoperative bariatric care). Our study reports initial outcomes (ie, first follow-up period at 1.5 years after surgery) of an RCT with a follow-up period extending to 3 years after surgery. Primary hypotheses were that the tele-CBT group would report greater weight loss and greater improvements in psychosocial functioning (ie, binge eating, emotional eating, depression, and anxiety) compared with the control group.

## Methods

This RCT was approved by the University Health Network Research Ethics Board in Toronto, Canada, which was the research ethics board of record. Informed consent was obtained from all individual participants included in the study. The Consolidated Standards of Reporting Trials (CONSORT) reporting guideline was used when writing this report.

### Study Setting

Patients were recruited from the Toronto Western Hospital Bariatric Centre of Excellence (TWH-BCOE), Humber River Hospital Bariatric Surgery Program (HRH-BSP), and Ottawa Hospital Bariatric Centre of Excellence (TOH-BCOE) between February 2018 and December 2021. To be accepted into the TWH-BCOE, HRH-BSP, or TOH-BCOE, patients were required to be 18 years or older with a preoperative body mass index (BMI; calculated as weight in kilograms divided by height in meters squared) of 40 or greater or a BMI of 35 or greater with at least 1 obesity-related comorbidity. All patients underwent a Roux-en-Y gastric bypass or a sleeve gastrectomy. Patients were eligible to participate in the study if they were 1 year after bariatric surgery, fluent in English, and had access to a telephone and a computer with internet connection to complete questionnaires. Study exclusion criteria included active suicidal ideation or poorly controlled severe psychiatric illness (eg, current psychosis) that would preclude a patient’s ability to engage in tele-CBT sessions.

### Study Procedures

The study used a 2-group RCT design. Patients were screened by the research coordinator (S.E.L.) to determine their study eligibility. Patients self-reported race and ethnicity in a single question during the baseline questionnaire. Options were Aboriginal (eg, Inuit, Metis, or North American Indian), Arab or West Asian (eg, Armenian, Egyptian, Iranian, or Lebanese), Black (eg, African, Haitian, Jamaican, or Somali), East Asian (eg, Chinese, Japanese, or Korean), Latin American, South Asian, Southeast Asian, White, or other (patients typed in their own responses). Race and ethnicity were assessed given that most bariatric surgery programs have predominantly White populations (approximately 80%). We would possibly like to assess in a subanalysis whether race and ethnicity may be a predictor of treatment response for our intervention. This would also help to identify if there are any gaps in our intervention to inform future studies on better ways to adapt the intervention for more marginalized racial and ethnic groups. Participants completed a series of questionnaires at baseline (1 year after surgery; time 1 [T1]) and were subsequently randomized 1:1 to the tele-CBT or treatment-as-usual control group. Open randomization was used, with permuted blocks of sizes 4 and 6 and stratification by site. The randomization sequence was generated in R statistical software version 3.4.3 (R Project for Statistical Computing) and implemented initially through a custom web-based application, then switched to a manual reveal of allocations for the final few months of participant randomization given that the application was no longer supported. This was an unblinded study. Participants randomized to the control group received standard bariatric care, which included access to and ongoing monitoring by their multidisciplinary bariatric surgery team, including a dietitian, social worker, and psychologist, psychiatrist, or both. Participants randomized to the tele-CBT group received the intervention described subsequently. All participants completed the same questionnaires online using Qualtrics survey software version February 2018 to December 2021 (Qualtrics) after the intervention (approximately 15 months after surgery; time 2 [T2]) and at 3-month follow-up (approximately 18 months after surgery; hereafter, *follow-up*; time 3 [T3]). The total time interval was 12 weeks between the baseline and postintervention questionnaire and 24 weeks between the baseline and follow-up questionnaire. For more information, please see Supplement 1 for the trial protocol.

### Intervention

The full tele-CBT protocol was detailed previously.^15,16,17^ It consisted of 6 weekly 1-hour sessions and a seventh 1-hour booster session delivered 1 month later. Sessions were conducted by 5 clinical psychology doctoral students with experience in the assessment and treatment of patients who underwent bariatric surgery. Study therapists received clinical supervision from a registered clinical psychologist (S.E.C.) and had biweekly group supervision.

The tele-CBT intervention includes setting goals, identifying and planning for difficult eating scenarios, planning pleasurable activities as an alternative to overeating, reducing vulnerability to overeating by challenging negative thoughts and solving problems, and scheduling healthy meals and snacks at regular intervals. Participants completed worksheets between sessions and implemented skills taught during sessions. The final booster session allowed participants to review skills they had learned, develop a relapse-prevention plan, and troubleshoot any issues that arose.

### Study Measures

#### Primary Outcome: Weight

Participants weighed themselves in pounds or kilograms and sent in an image of their scale to report their weight at baseline, after intervention, and at follow-up. Postoperative percentage total weight loss was calculated using the following formula: percentage total weight loss = ([baseline weight in kilograms − postintervention or follow-up weight in kilograms]/baseline weight in kilograms) × 100. Weight outcomes were calculated using participant-reported weight at baseline rather than presurgery weight to determine whether the tele-CBT intervention was efficacious in affecting weight outcomes and to eliminate the surgery itself as a factor for weight loss.

#### Secondary Outcome: Disordered Eating

Disordered eating was assessed using the Binge Eating Scale (BES)^18,19^ and Emotional Eating Scale (EES).^20^ The BES is a 16-item self-report measure designed specifically for use with individuals with obesity and assesses the presence of binge-eating characteristics indicative of an eating disorder. Scores on the BES range from 0 to 46, with moderate and severe levels of binge eating corresponding to cutoff scores of 18 and 27, respectively. At a cutoff point of greater than 17, the BES has a sensitivity of 94% and a specificity of 75%.^19^ The EES is a 25-item self-report measure that assesses a person’s tendency to cope with negative affect through eating. The scale ranges from 0 (no desire) to 4 (overwhelming urge) and consists of questions that ask participants to rate the intensity of their urges to eat in response to 25 emotions. The EES comprises 3 subscales that reflect eating in response to anger or frustration, anxiety, and depression. Both scales have been used in bariatric surgery populations to detect changes in eating psychopathology after surgery.^15,20^

#### Secondary Outcome: Psychological Distress

Psychological distress was assessed using the Patient Health Questionnaire 9-item scale (PHQ-9)^21^ and Generalized Anxiety Disorder 7-item scale (GAD-7).^22,23^ Scores on the PHQ-9 range from 0 to 27, with mild, moderate, moderately severe, and severe levels of depressive symptoms corresponding to cutoff scores of 5, 10, 15, and 20, respectively. At a cutoff point of 10 or greater, the PHQ-9 has a sensitivity of 88% and a specificity of 88% for major depression.^21^ Scores on the GAD-7 range from 0 to 21, with mild, moderate, and severe levels of anxiety symptoms corresponding to cutoff scores of 5, 10, and 15, respectively. At a cutoff point of 10 or greater, the GAD-7 has a sensitivity of 89% and a specificity of 82%.^22^ Both measures have been used to assess treatment outcomes in patients who underwent bariatric surgery.^24,25,26^

### Sample Size and Power

In previous research on the impact of calorie-restricted dietary interventions that included CBT programs, weight loss was typically in the range of 7.5% to 10%.^27,28^ Discussion with bariatric surgeons has indicated that a difference of 5% in weight loss would be impressive enough to warrant a program to implement CBT. In our clinic data among 191 patients, we found that the mean weight at 2 years after surgery was 92 kg, the Pearson correlation between 1- and 2-year weights was 0.8, and the between-patient SD at 1-year and 2-year was 21 kg. A 5% difference in weight at 2 years (equivalently, a difference in 1- to 2-year weight change equal to 5% of the 2-year weight) is a clinically important difference and translates to approximately 4.5 kg. With a type I error rate of 5%, if the true difference in weights between control and tele-CBT groups at 2 years is 4.5 kg, a sample size of 124 individuals per group gives 80% power in an analysis of covariance with 1 year weight as the covariate. Anticipating up to 30% loss to follow-up or withdrawal between 1 and 2 years, it was determined that 175 participants would be enrolled per group.

### Statistical Analysis

Statistical analyses were performed using SPSS Statistics for Windows version 24.0 (IBM). Analyses adhered to the intent-to-treat principle. Participant characteristics were summarized using descriptive statistics, including means, SDs, frequencies, and proportions. The Shapiro-Wilk test was used to determine whether continuous outcomes were normally distributed. Nonnormally distributed outcomes were log(x) or log(x + 1) transformed for analysis. Linear mixed models with random intercepts were generated for each outcome variable, with fixed effects of group (control and tele-CBT), time (baseline, after intervention, and follow-up), and group-by-time interaction. For reporting, estimated means and SEs from mixed models were back-transformed to their original units as: exp(mean log) ± exp(mean log) × (exp(SE log)-1) or exp(mean log) − 1 ± exp(mean log) × (exp(SE log) − 1) for log(x) or log(x + 1) transformations, respectively (based on the Delta method^29^). Data were analyzed from January to February 2023.

#### Missing Data

Linear mixed models are generally robust against missing data.^30^ For each outcome, participants with at least 1 nonmissing outcome measure were entered into the linear mixed model. Imputation methods were not implemented.

#### Multiplicity

For the primary outcome (percentage total weight loss), a 2-sided *P* value < .05 was considered statistically significant. For secondary outcomes, 2-sided *P* values < .05/4 = 0.0125 were considered statistically significant after applying the Bonferroni correction for multiple testing.

## Results

### Participant Flow and Characteristics

Among 306 participants (mean [SD] age at baseline, 47.55 [9.98] years; 255 females [83.3%]; 10 Arab or West Asian [3.3%], 23 Black [7.5%], 9 Latin American [2.9%], 234 White [76.5%], and 19 other race or ethnicity [6.2%]), most participants were in a relationship (186 participants [60.8%]), had completed college or university (198 participants [64.7%]), and were employed full time (212 participants [69.3%]) (Table 1). The mean (SD) weight of participants at baseline was 93.78 (23.54) kg. There were no significant differences between groups at baseline for secondary outcome variables (see eTable in Supplement 2 for raw means).

**Table 1.** Participant Characteristics at Baseline

Characteristic	Participants, No. (%)		
	Tele-CBT (n = 152)	Control (n = 154)	Overall (N = 306)
Study site			
TWH-BCOE	130 (85.5)	131 (85.1)	261 (85.3)
HRH-BSP	17 (11.2)	17 (11.0)	34 (11.1)
TOH-BCOE	5 (3.3)	6 (3.9)	11 (3.6)
Age, mean (SD), y (n = 305)^a^	46.86 (10.33)	48.23 (9.61)	47.55 (9.98)
Weight, mean (SD) kg) (n = 305)^a^	98.23 (26.28)	89.35 (19.56)	93.78 (23.5)
BMI, mean (SD) (n = 303)^a^	34.77 (8.46)	32.20 (6.06	33.48 (7.46)
Surgery type, Roux-en-Y	117 (77.0)	131 (85.1)	248 (81)
Sex			
Female	126 (82.9)	129 (83.8)	255 (83.3)
Male	26 (17.1)	24 (15.6)	50 (16.3)
Missing	0	1 (0.6)	1 (0.3)
Race and ethnicity			
Aboriginal	2 (1.3)	1 (0.6)	3 (1)
Arab or West Asian	3 (2.0)	7 (4.5)	10 (3.3)
Black	12 (7.9)	11 (7.1)	23 (7.5)
East Asian	0	1 (0.6)	1 (0.3)
Latin American	4 (2.6)	5 (3.2)	9 (2.9)
South Asian	2 (1.3)	1 (0.6)	3 (1.0)
Southeast Asian	2 (1.3)	1 (0.6)	3 (1.0)
White	114 (75.0)	120 (77.9)	234 (76.5)
Other	12 (7.9)	7 (4.5)	19 (6.2)
Missing	1 (0.7)	0	1 (0.3)
Relationship status			
Married or common-law	89 (58.6)	97 (63.0)	186 (60.8)
Divorced or separated	22 (14.5)	20 (13.0)	42 (13.7)
Single	37 (24.3)	37 (24.0)	74 (24.2)
Widowed	3 (2.0)	0	3 (1.0)
Missing	1 (0.7)	0	1 (0.3)
Occupational status			
Full time	98 (64.5)	114 (74.0)	212 (69.3)
Part time	12 (7.9)	10 (6.5)	22 (7.2)
Retired	14 (9.2)	12 (7.8)	26 (8.5)
Social assistance	1 (0.7)	1 (0.6)	2 (0.7)
Disability	17 (11.2)	8 (5.2)	25 (8.2)
Unemployed	9 (5.9)	9 (5.8)	18 (5.9)
Missing	1 (0.7)	0	1 (0.3)
Education			
Some high school	7 (4.6)	1 (0.6)	8 (2.6)
High school graduate	12 (7.9)	15 (9.7)	27 (8.8)
Some college or university	36 (23.7)	34 (22.1)	70 (22.9)
College or university graduate	94 (61.8)	104 (67.5)	198 (64.7)
Missing	3 (2.0)	0	3 (1.0)

Abbreviations: BMI, body mass index (calculated as weight in kilograms divided by height in meters squared); HRH-BSP, Humber River Hospital Bariatric Surgery Program; TOH-BCOE, Ottawa Hospital Bariatric Centre of Excellence; TWH-BCOE, Toronto Western Hospital Bariatric Centre of Excellence.

^a^ Baseline was 1 year after surgery.

**Figure 1.**
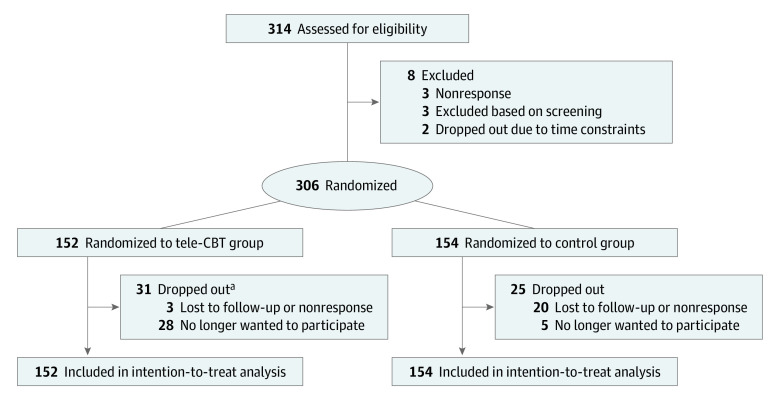
Study Flowchart Tele-CBT indicates telephone-based cognitive behavioral therapy. ^a^Of all dropouts, 23 patients dropped out during tele-CBT treatment.

Participant flow is detailed in the flowchart in Figure 1. Of 314 patients who consented to participate, 306 individuals (97.5%) completed the baseline questionnaire and were randomized to the tele-CBT (152 participants) or control (154 participants) group. Of the remaining participants, 3 individuals did not respond to calls or emails, 2 individuals dropped out due to time constraints, and 3 individuals were excluded due to screening results. After randomization, 56 participants (18.3%) discontinued from the study, with 23 participants being lost to follow-up and the remaining 33 participants choosing to discontinue due to time constraints, not benefiting from the study, or general disinterest to continue. Of these 56 participants, 31 participants were in the tele-CBT group; 23 of these participants dropped out during the tele-CBT intervention. Of 152 participants in the tele-CBT intention-to-treat group, 127 individuals (83.6%) completed the tele-CBT intervention. A total of 123 patients (80.9%) completed both treatment and 1.5-year postsurgery follow-up. For the actual enrollment of 152 and 154 participants, the statistical power of the final sample was 87.6%.

### Comparison of Tele-CBT and Control Groups on Outcomes Across Time

**Figure 2.**
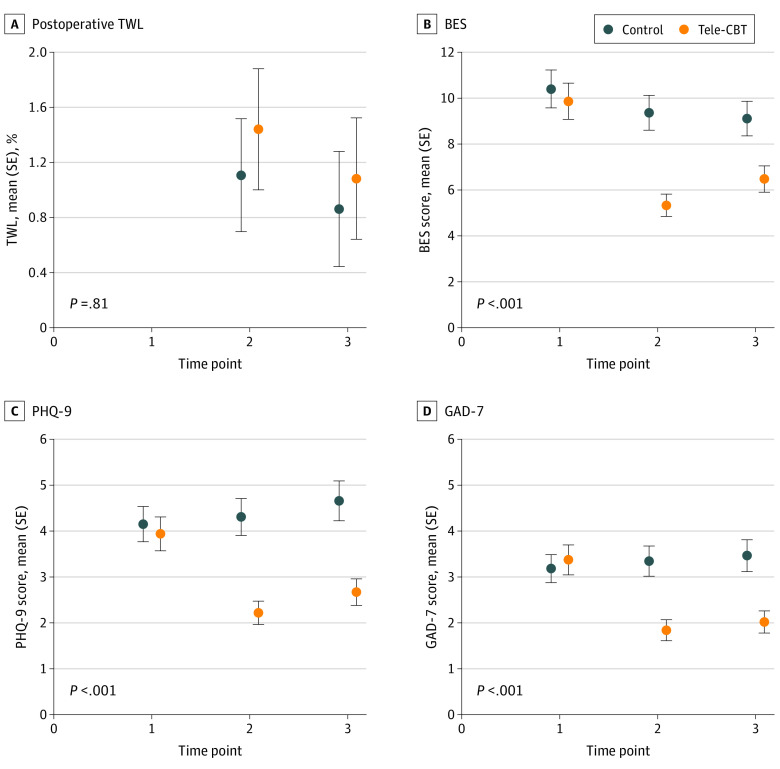
Estimated Mean Outcomes From Linear Mixed Models Times 1, 2, and 3 refer to baseline (1 year after surgery), after intervention (approximately 15 months after surgery), and 3-month follow-up (approximately 18 months after surgery), respectively. *P* values refer to the group-by-time interaction. BES indicates Binge Eating Scale; GAD-7, Generalized Anxiety Disorder-7 item scale; PHQ-9, Patient Health Questionnaire 9-item scale; SE, standard error; tele-CBT, telephone-based cognitive behavioral therapy; TWL, total weight loss.

Estimated mean scores and SEs from linear mixed models for each clinical outcome by group at baseline, after intervention, and at follow-up are presented in Table 2 and Figure 2. The group-by-time interaction for the primary outcome of percentage total weight loss was not significant (*F*_1,160.61_ = 2.09; *P* = .15). Participants in the tele-CBT group attained a mean (SE) weight loss of 1.44% (0.44%) and 1.08% (0.44%) at T2 and T3, respectively. In comparison, participants in the control group attained a mean (SE) weight loss of 1.11% (0.41%) and 0.86% (0.42%) at T2 and T3, respectively. There was a significant group-by-time interaction for disordered eating and psychological distress outcomes. Specifically, there was a significant decrease in mean BES (*F*_2,527.32_ = 18.73; *P* < .001), EES total (*F*_2,530.67_ = 10.83; *P* < .001), PHQ-9 (*F*_2,529.93_ = 17.74; *P* < .001), and GAD-7 (*F*_2,535.16_ = 15.29; *P* < .001) scores for the tele-CBT group indicative of improvement from baseline to after intervention and follow-up, whereas scores for the control group stayed the same or increased over the same period (Figure 2; eFigure in Supplement 2).

**Table 2.** Clinical Variables by Study Group Over Time

Measure	Mean (SE) (N = 306)^a^					
	Baseline^b^		After intervention^b^		Follow-up^b^	
	Tele-CBT	Control	Tele-CBT	Control	Tele-CBT	Control
Percentage TWL	NA	NA	1.44 (0.44)	1.11 (0.41)	1.08 (0.44)	0.86 (0.42)
BES	9.87 (0.79)	10.40 (0.83)	5.33 (0.49)	9.37 (0.76)	6.48 (0.57)	9.11 (0.76)
EES total	46.80 (1.85)	45.29 (1.79)	37.71 (1.58)	44.29 (1.80)	39.69 (1.66)	45.34 (1.84)
PHQ-9	3.94 (0.37)	4.15 (0.38)	2.22 (0.25)	4.31 (0.40)	2.67 (0.29)	4.66 (0.43)
GAD-7	3.37 (0.33)	3.18 (0.31)	1.84 (0.23)	3.34 (0.33)	2.02 (0.24)	3.46 (0.35)

Abbreviations: BES, Binge Eating Scale; EES, Emotional Eating Scale; GAD-7, Generalized Anxiety Disorders 7-item scale; NA, not applicable; PHQ-9, Patient Health Questionnaire 9-item scale; SE, standard error; TWL, total weight loss.

^a^ All outcomes were nonnormally distributed and log(x) transformed for analysis. For reporting, estimated means and SEs from mixed models were back-transformed to their original units as: exp (mean log) ± exp(mean log) × (exp(SE log) − 1) or exp(mean log) − 1 ± exp(mean log) × (exp(SE log) − 1) for log(x) or log(x + 1) transformations, respectively.

^b^ Baseline was 1 year after surgery. After intervention was approximately 15 months after surgery. Follow-up was approximately 18 months after surgery.

## Discussion

Disordered eating and psychological distress are well-documented risk factors associated with weight regain after bariatric surgery. This multisite RCT sought to test the efficacy of a tele-CBT intervention targeting these risk factors 1 year after surgery in weight loss outcomes 1.5 years after surgery. To our knowledge, this is the largest RCT examining psychosocial interventions to support patients after bariatric surgery. Contrary to our hypothesis, the tele-CBT and control groups did not significantly differ in percentage weight loss at 1.5 years after surgery. However, the tele-CBT group did demonstrate significant improvements in binge eating, emotional eating, and depressive and anxiety symptoms immediately after the intervention, and these improvements were maintained at 3 months after the intervention (1.5 years after bariatric surgery).

The study showed a high engagement rate in tele-CBT at 1 year after surgery, with 123 patients (80.9%) completing both the treatment and the 1.5-year postsurgery follow-up, and a high engagement rate of the intervention itself, with a completion rate of 127 participants (83.6%) for tele-CBT. Given the high attrition rates related to follow-up after bariatric surgery and the expansion of virtual psychosocial interventions for patients who underwent bariatric surgery, the high acceptability of treatment and retention of patients in this telephone-delivered psychological intervention is noteworthy.^31,32^

Although tele-CBT did not significantly improve weight loss outcomes at 1.5 years after surgery in comparison with standard bariatric surgery aftercare, it did significantly improve disordered eating and psychological distress. Tele-CBT and control groups had BES, PHQ-9, and GAD-7 scores below the threshold of clinical significance. Despite this outcome, the tele-CBT group reported statistically significant improvement in these variables compared with the control group across time. Postoperative disordered eating behaviors, such as loss of control over eating, were found to be associated with attenuated weight loss outcomes and weight regain after bariatric surgery.^33,34,35^ Rates of loss of control over eating are estimated to range from 10% to 39% at 2-year postsurgery follow-up.^35,36^ Therefore, results of this multisite RCT provide further evidence for the efficacy of this intervention in treating disordered eating after bariatric surgery, which could potentially improve weight loss and mitigate weight regain long term.

The tele-CBT intervention also improved anxiety and depressive symptoms. A deterioration in postsurgery mental health–related quality of life has been negatively associated with increased depressive and anxiety symptoms.^24,37^ A longitudinal study assessing depressive symptoms and weight outcomes over a 7-year period reported that the trajectory characterized by initial reductions in depressive symptoms was associated with the greatest weight loss.^38^ The group recommended the first postoperative year as a key intervention period, noting that early improvement in depression may be associated with longer-term weight outcomes.^38^ Furthermore, 7-year data from the Longitudinal Assessment of Bariatric Surgery (LABS) Consortium^37^ suggest that having a postoperative mood disorder may be associated with weight regain.

### Strengths and Limitations

Study strengths included the multisite RCT study design, standard postoperative bariatric care control group, large sample size, high retention rate, and psychometrically sound outcome measures that have been commonly used in bariatric populations. This study also has several limitations. These included the short-term postintervention follow-up, which may have limited our ability to observe treatment effects on weight loss outcomes. Additionally, energy intake and expenditure were not collected and were not part of this intervention, which may explain why there were no changes in short-term weight loss outcomes given that no caloric-restricted dietary intervention was used. Data collection extending to 3 years after surgery is still in progress and may help determine whether improvements in disordered eating and psychological distress are maintained over time and lead to better weight outcomes 3 years after surgery. Moreover, patients at 1 year after surgery in this RCT may still be too early in their postbariatric surgery journey to experience significant risks of weight regain, and future studies could examine the effects of tele-CBT delivered beyond the first 2 years after surgery. Additionally, bariatric centers of excellence included in this multisite RCT offered routine postsurgery care in which behavioral health support from a dietitian, social worker, and psychologist, psychiatrist, or both was part of the care provided by the interprofessional team. The standard postoperative model of care received by the control group may not be the standard in all countries and practice contexts, and this additional integrated support may have provided sufficient support to attenuate weight loss outcome differences between tele-CBT and control groups. Nonetheless, it is noteworthy that significant differences were observed in disordered eating and psychological distress symptoms between groups despite the standard care control groups’ access to behavioral health supports. Future studies should examine the efficacy of this intervention in other contexts with different models of care. The intervention could be more scalable by developing an online training program to teach bariatric clinicians how to deliver the CBT protocol and by developing a user-friendly patient workbook to share with patients who underwent bariatric surgery. Previous research has found group CBT to be associated with improved disordered eating among patients who underwent bariatric surgery,^13^ and that may be another avenue for increasing access to this intervention. Additionally, study inclusion criteria did not require patients to have current disordered eating or psychological distress (eg, some patients who experienced preoperative binge eating set goals of developing habits and learning coping skills to prevent the recurrence of binge eating), which may underestimate the potential effect of tele-CBT on these psychosocial and weight loss outcomes. Future studies should recruit patients with clinically significant depression and binge eating to determine the efficacy of the intervention in populations at higher risk of weight regain.

## Conclusions

Although tele-CBT did not affect short-term weight outcomes, the intervention demonstrated significant reductions in eating psychopathology, depressive symptoms, and anxiety symptoms after bariatric surgery in the largest RCT to date, to our knowledge. These findings support the Canadian Adult Obesity Clinical Practice Guidelines, which shift the focus of obesity care to health and quality of life outcomes rather than weight loss alone. Our tele-CBT intervention had a high retention rate as a virtual intervention to support patients after bariatric surgery and may have the potential to address weight regain long term by mitigating disordered eating and mental health risks factors.

## Notes

Supplement 1.
Trial Protocol and Statistical Analysis Plan

Supplement 2. **eTable 1.** Raw Means for Clinical Variables **eFigure.** Estimated Mean Outcomes From a Linear Mixed Model Comparing Emotional Eating Scale Total Score

Supplement 3.
Data Sharing Statement

## References

[1] World Health Organization. Obesity and overweigh. Accessed July 7, 2023. https://www.who.int/news-room/fact-sheets/detail/obesity-and-overweight

[2] Adams TD, Davidson LE, Litwin SE, . Weight and metabolic outcomes 12 years after gastric bypass. N Engl J Med. 2017;377(12):1143-1155. doi:10.1056/NEJMoa1700459 28930514 PMC5737957

[3] Eisenberg D, Shikora SA, Aarts E, . 2022 American Society for Metabolic and Bariatric Surgery (ASMBS) and International Federation for the Surgery of Obesity and Metabolic Disorders (IFSO): indications for metabolic and bariatric surgery. Surg Obes Relat Dis. 2022;18(12):1345-1356. doi:10.1016/j.soard.2022.08.013 36280539

[4] Sjöström L, Narbro K, Sjöström CD, ; Swedish Obese Subjects Study. Effects of bariatric surgery on mortality in Swedish obese subjects. N Engl J Med. 2007;357(8):741-752. doi:10.1056/NEJMoa066254 17715408

[5] Arterburn DE, Olsen MK, Smith VA, . Association between bariatric surgery and long-term survival. JAMA. 2015;313(1):62-70. doi:10.1001/jama.2014.16968 25562267

[6] Noria SF, Shelby RD, Atkins KD, Nguyen NT, Gadde KM. Weight regain after bariatric surgery: scope of the problem, causes, prevention, and treatment. Curr Diab Rep. 2023;23(3):31-42. doi:10.1007/s11892-023-01498-z 36752995 PMC9906605

[7] Athanasiadis DI, Martin A, Kapsampelis P, Monfared S, Stefanidis D. Factors associated with weight regain post-bariatric surgery: a systematic review. Surg Endosc. 2021;35(8):4069-4084. doi:10.1007/s00464-021-08329-w 33650001

[8] Mauro MFFP, Papelbaum M, Brasil MAA, . Is weight regain after bariatric surgery associated with psychiatric comorbidity: a systematic review and meta-analysis. Obes Rev. 2019;20(10):1413-1425. doi:10.1111/obr.12907 31322316

[9] Alyahya RA, Alnujaidi MA. Prevalence and outcomes of depression after bariatric surgery: a systematic review and meta-analysis. Cureus. 2022;14(6):e25651. doi:10.7759/cureus.25651 35784972 PMC9249077

[10] Debédat J, Sokolovska N, Coupaye M, . Long-term relapse of type 2 diabetes after Roux-en-Y gastric bypass: prediction and clinical relevance. Diabetes Care. 2018;41(10):2086-2095. doi:10.2337/dc18-0567 30082327

[11] King WC, Hinerman AS, Belle SH, Wahed AS, Courcoulas AP. Comparison of the performance of common measures of weight regain after bariatric surgery for association with clinical outcomes. JAMA. 2018;320(15):1560-1569. doi:10.1001/jama.2018.14433 30326125 PMC6233795

[12] Conceição EM, Goldschmidt A. Disordered eating after bariatric surgery: clinical aspects, impact on outcomes, and intervention strategies. Curr Opin Psychiatry. 2019;32(6):504-509. doi:10.1097/YCO.0000000000000549 31343419 PMC6768715

[13] David LA, Sijercic I, Cassin SE. Preoperative and post-operative psychosocial interventions for bariatric surgery patients: a systematic review. Obes Rev. 2020;21(4):e12926. doi:10.1111/obr.12926 31970925

[14] Newman AK, Herbozo S, Russell A, . Psychosocial interventions to reduce eating pathology in bariatric surgery patients: a systematic review. J Behav Med. 2021;44(3):421-436. doi:10.1007/s10865-021-00201-5 33580454

[15] Sockalingam S, Leung SE, Hawa R, . Telephone-based cognitive behavioural therapy for female patients 1-year post-bariatric surgery: a pilot study. Obes Res Clin Pract. 2019;13(5):499-504. doi:10.1016/j.orcp.2019.07.003 31409544

[16] Cassin SE, Sockalingam S, Du C, Wnuk S, Hawa R, Parikh SV. A pilot randomized controlled trial of telephone-based cognitive behavioural therapy for preoperative bariatric surgery patients. Behav Res Ther. 2016;80:17-22. doi:10.1016/j.brat.2016.03.001 26990279 PMC5468091

[17] Cassin SE, Sockalingam S, Wnuk S, . Cognitive behavioral therapy for bariatric surgery patients: preliminary evidence for feasibility, acceptability, and effectiveness. Cogn Behav Pract. 2013;20(4):529-543. doi:10.1016/j.cbpra.2012.10.002

[18] Gormally J, Black S, Daston S, Rardin D. The assessment of binge eating severity among obese persons. Addict Behav. 1982;7(1):47-55. doi:10.1016/0306-4603(82)90024-7 7080884

[19] Hood MM, Grupski AE, Hall BJ, Ivan I, Corsica J. Factor structure and predictive utility of the Binge Eating Scale in bariatric surgery candidates. Surg Obes Relat Dis. 2013;9(6):942-948. doi:10.1016/j.soard.2012.06.013 22963818 PMC4874331

[20] Arnow B, Kenardy J, Agras WS. The Emotional Eating Scale: the development of a measure to assess coping with negative affect by eating. Int J Eat Disord. 1995;18(1):79-90. doi:10.1002/1098-108X(199507)18:1<79::AID-EAT2260180109>3.0.CO;2-V 7670446

[21] Kroenke K, Spitzer RL, Williams JB. The PHQ-9: validity of a brief depression severity measure. J Gen Intern Med. 2001;16(9):606-613. doi:10.1046/j.1525-1497.2001.016009606.x 11556941 PMC1495268

[22] Spitzer RL, Kroenke K, Williams JB, Löwe B. A brief measure for assessing generalized anxiety disorder: the GAD-7. Arch Intern Med. 2006;166(10):1092-1097. doi:10.1001/archinte.166.10.1092 16717171

[23] Kroenke K, Spitzer RL, Williams JB, Monahan PO, Löwe B. Anxiety disorders in primary care: prevalence, impairment, comorbidity, and detection. Ann Intern Med. 2007;146(5):317-325. doi:10.7326/0003-4819-146-5-200703060-00004 17339617

[24] Youssef A, Keown-Stoneman C, Maunder R, . Differences in physical and mental health-related quality of life outcomes 3 years after bariatric surgery: a group-based trajectory analysis. Surg Obes Relat Dis. 2020;16(11):1837-1849. doi:10.1016/j.soard.2020.06.014 32737009

[25] Cassin S, Sockalingam S, Hawa R, . Psychometric properties of the Patient Health Questionnaire (PHQ-9) as a depression screening tool for bariatric surgery candidates. Psychosomatics. 2013;54(4):352-358. doi:10.1016/j.psym.2012.08.010 23274006

[26] Sockalingam S, Hawa R, Wnuk S, . Psychosocial predictors of quality of life and weight loss two years after bariatric surgery: results from the Toronto Bari-PSYCH study. Gen Hosp Psychiatry. 2017;47:7-13. doi:10.1016/j.genhosppsych.2017.04.005 28807141

[27] Cooper Z, Doll HA, Hawker DM, . Testing a new cognitive behavioural treatment for obesity: a randomized controlled trial with three-year follow-up. Behav Res Ther. 2010;48(8):706-713. doi:10.1016/j.brat.2010.03.008 20691328 PMC2923743

[28] Werrij MQ, Jansen A, Mulkens S, Elgersma HJ, Ament AJ, Hospers HJ. Adding cognitive therapy to dietetic treatment is associated with less relapse in obesity. J Psychosom Res. 2009;67(4):315-324. doi:10.1016/j.jpsychores.2008.12.011 19773024

[29] Casella G, Berger RL. Statistical Inference. 2nd ed. Wadsworth Group; 2002.

[30] Diggle PJ, Heagerty PK, Liang KY, Zeger SL. Analysis of Longitudinal Data. 2nd ed. Oxford University Press; 2013.

[31] Lohnberg JA, Salcido L, Frayne S, . Rapid conversion to virtual obesity care in COVID-19: impact on patient care, interdisciplinary collaboration, and training. Obes Sci Pract. 2021;8(1):131-136. doi:10.1002/osp4.550 34540265 PMC8441727

[32] Sockalingam S, Leung SE, Cassin SE. The impact of coronavirus disease 2019 on bariatric surgery: redefining psychosocial care. Obesity (Silver Spring). 2020;28(6):1010-1012. doi:10.1002/oby.22836 32294297 PMC7262315

[33] Nasirzadeh Y, Kantarovich K, Wnuk S, . Binge eating, loss of control over eating, emotional eating, and night eating after bariatric surgery: results from the Toronto Bari-PSYCH cohort study. Obes Surg. 2018;28(7):2032-2039. doi:10.1007/s11695-018-3137-8 29411241

[34] Devlin MJ, King WC, Kalarchian MA, . Eating pathology and associations with long-term changes in weight and quality of life in the longitudinal assessment of bariatric surgery study. Int J Eat Disord. 2018;51(12):1322-1330. doi:10.1002/eat.22979 30520527 PMC6876117

[35] Conceição EM, Mitchell JE, Pinto-Bastos A, Arrojado F, Brandão I, Machado PPP. Stability of problematic eating behaviors and weight loss trajectories after bariatric surgery: a longitudinal observational study. Surg Obes Relat Dis. 2017;13(6):1063-1070. doi:10.1016/j.soard.2016.12.006 28209532

[36] White MA, Kalarchian MA, Masheb RM, Marcus MD, Grilo CM. Loss of control over eating predicts outcomes in bariatric surgery patients: a prospective, 24-month follow-up study. J Clin Psychiatry. 2010;71(2):175-184. doi:10.4088/JCP.08m04328blu 19852902 PMC2831110

[37] Kalarchian MA, King WC, Devlin MJ, . Mental disorders and weight change in a prospective study of bariatric surgery patients: 7 years of follow-up. Surg Obes Relat Dis. 2019;15(5):739-748. doi:10.1016/j.soard.2019.01.008 30826244 PMC7045720

[38] Smith KE, Mason TB, Cao L, . Trajectories of depressive symptoms and relationships with weight loss in the seven years after bariatric surgery. Obes Res Clin Pract. 2020;14(5):456-461. doi:10.1016/j.orcp.2020.08.007 32933863 PMC8320367

