# Supplementary material for: Efficacy of Telephone-Based Cognitive Behavioral Therapy for Weight Loss, Disordered Eating, and Psychological Distress After Bariatric Surgery: A Randomized Clinical Trial
Source: JAMA Netw Open. 2023 Aug 3;6(8):e2327099. doi: 10.1001/jamanetworkopen.2023.27099 (PMC10401302; doi:10.1001/jamanetworkopen.2023.27099)
Supplement: Supplement 1. — Trial Protocol and Statistical Analysis Plan [file jamanetwopen-e2327099-s001.pdf]

Telephone-Based Cognitive Behavioural Therapy for Post-Operative Bariatric Surgery Patients: A Randomized Controlled Trial

Protocol November 14, 2017

Investigators:
Dr. Sanjeev Sockalingam (Nominated PI)
Dr. Stephanie Cassin (co-PI)
Dr. Raed Hawa
Dr. Susan Wnuk
Dr. Timothy Jackson
Dr. Lorraine Lipscombe
Dr. Allan Okrainec
Dr. George Tomlinson
Dr. David Urbach

Rationale and Background:

Obesity is Canada's fastest growing public health problem.[1] Over the past 20 years, obesity has surpassed smoking as the leading cause of preventable death in developed countries, and has surpassed under-nutrition and infectious disease as the most significant predictor of ill health and disease.[2,3] The World Health Organization defines obesity as a body mass index (BMI) of 30 kg/m2 or greater: Class I obesity (BMI 30-34.9 kg/m2), Class II obesity (BMI 35-39.9 kg/m2), and Class III or "extreme" obesity (BMI > 40 kg/m2).[4] Of the 1 in 4 Canadians with obesity,[5] approximately 1.07 million Canadians have the most rapidly increasing Class III obesity, and an additional 2.0 million have class II obesity.[6,7] Obesity is associated with significant medical comorbidities and has a high rate of mortality.[8-10]

Empirically supported treatment options for obesity include pharmacotherapy, multi-component behavioural lifestyle interventions, and surgical interventions.[11] Bariatric surgery, a procedure that restricts the stomach's capacity for food and/or limits the absorption of food, is the most effective treatment for patients with Class II and Class III obesity. Major clinical guidelines recommend bariatric surgery for individuals with a BMI > 40 kg/m^2, and those with a BMI > 35 kg/m^2 and significant obesity-related medical comorbidities. [11] Accordingly, the demand for bariatric surgery has been rapidly increasing.[12,13] The Ontario Ministry of Health and Long Term Care responded by investing $75 million to increase bariatric surgery capacity by 500% over 3 years and created the Ontario Bariatric Network (OBN).

The most frequently performed bariatric surgery is the Roux-en-Y gastric bypass.[12,14,15] Patients who undergo this procedure lose an average of 20 to 30% of their total body weight,[16,17] and experience dramatic improvements or complete resolution of many of their medical comorbidities including type 2 diabetes mellitus (T2DM: 77%), hypertension (62%), and obstructive sleep apnea (86%).[18,19] However, **weight change trajectories are highly variable**.[20] Approximately 50% of patients experience some weight regain in the first 2 years,[21] and 24% experience weight regain that is considerable relative to their overall weight loss by 3 years post-

surgery.[22] Moreover, **weight regain is associated with relapse of obesity-related comorbidities**.[22,23]

While bariatric surgery is effective in reducing weight and improving many obesity related comorbidities, it **does not directly target the underlying behavioural and psychological factors that potentially contribute to the development and maintenance of obesity**. Psychosocial factors play an important role in long-term outcomes.[24,25] High rates of psychiatric comorbidity have been documented in bariatric patients.[26] Post-operative eating pathology and depression are among the most consistent negative predictors of weight loss outcomes.[24,25] These factors also predispose to obesity, thus, they need to be targeted alongside bariatric surgery to prevent relapse.

Psychosocial interventions are increasingly being recommended in the clinical management of bariatric patients.[21,27,28] Although no "best practices" have been established, review papers and metaanalyses suggest that Cognitive Behavioural Therapy (CBT) targeting disordered eating and psychological factors offers the greatest promise.[27,28] Cognitive behavioural therapy incorporates both behavioural interventions (e.g., food monitoring, weekly weighing, goal setting, behavioural activation, stimulus control, environmental contingencies) and cognitive interventions (e.g., identifying, challenging, and altering counterproductive thoughts). Major clinical guidelines recommend CBT as a first-line treatment for many of the psychological disorders that are prevalent among bariatric surgery patients, including depression[29] and binge eating disorder,[30] and empirical research also supports the efficacy of CBT for weight management.[31-34]

A number of uncontrolled trials and randomized controlled trials have examined CBT specifically in bariatric surgery populations. A systematic review and meta-analysis of randomized and unrandomized trials reported significant improvements in binge eating, depression, anxiety, and quality of life following CBT interventions in bariatric populations.[28] A more recent review examining psychosocial interventions pre and post-bariatric surgery arrived at the same conclusion, but further noted that the **evidence was stronger for post-operative interventions**.[27] Small sample sizes and high attrition rates were noted as common limitations in both reviews.[27,28] Of note, the psychosocial interventions examined to date have relied exclusively on face-to-face treatment sessions, with the exception of two studies that incorporated the use of telephone sessions and recommended further exploration of telephone-based interventions in bariatric surgery populations.[35,36]

Why use Telephone-Based Interventions?: Travelling for appointments is difficult for bariatric patients who often reside great distances from bariatric programs and have mobility challenges secondary to obesity.[37,38] At the Toronto Western Hospital Bariatric Surgery Program (TWH-BSP), patients travel an average of 138.5 kilometers (range: 2.0 to 1734.0 kilometers) to the program,[39] so many cannot feasibly attend weekly therapy sessions. Approximately 70% of bariatric patients adhere to in person 12-month post-surgery appointments in our program, and 50% adhere to in-person 24- month appointments.[39] Travel distance is inversely associated with post-operative attrition, further reinforcing the need for psychosocial treatment modalities that can overcome this barrier.[40,41] Novel methods for delivering CBT, such as Tele-CBT, obviate the need for travel and improve access because treatments can be delivered during the evenings and weekends, eliminating the need to take time off work or find childcare.[42] A systematic review reported positive outcomes in 83% of studies examining the impact of telephone-based interventions on dietary behaviours.[43] Tele-CBT has been shown efficacious in treating various forms of psychopathology that are common among bariatric surgery patients, including

depression and binge eating disorder.[44-47] In addition, patients who receive Tele-CBT report comparable levels of treatment satisfaction to those who receive face-to-face CBT,[48] and they have lower rates of attrition,[49] suggesting that Tele-CBT is an acceptable form of treatment. A recent Technical Brief prepared for the U.S. Department of Health, which reviewed the findings from[50] systematic reviews of telehealth interventions, concluded that the most consistent benefit has been reported when telehealth has been used to counsel patients with chronic health conditions or to provide psychotherapy as part of behavioural health.[51] Although no randomized controlled trials conducted to date have examined the efficacy of telephone-based interventions for the management of severe obesity, all indications suggest that Tele-CBT has the potential to improve patient outcomes following bariatric surgery.

Pilot Study: Our team developed a manual-based Tele-CBT protocol[52] and conducted a series of pilot studies to examine the feasibility, acceptability, and efficacy of the protocol.[52-54] We first conducted a small prepilot study (*N* = 8; 2 pre-op, 6 post-op; 6 face-to-face, 2 telephone) to validate the manual and examine the feasibility and acceptability of the protocol.[52] Patients reported a high level of satisfaction with CBT, and experienced improvements in binge eating, loss of control over eating, and emotional eating immediately following the intervention. Minor modifications were made to the protocol based on client and clinician feedback. We subsequently conducted a pilot RCT in which bariatric patients (*N* = 47) received Tele-CBT either pre-operatively,[53] or 6 months post-operatively.[54] Participants in both groups reported significant improvements in binge eating, emotional eating, depression, and anxiety following the intervention, with large to very large effect sizes reported from pre- to post-treatment on each measure. However, participants favoured a post-operative intervention and recommended that it be offered following the "honeymoon period" of weight loss. Of note, this patient feedback corroborates the recommendation made by a recent review paper that psychosocial interventions be delivered in the post-operative period, following the "honeymoon period" but prior to significant weight regain.[27] Qualitative feedback from our patients indicates that the majority believe 1 year postsurgery to be the ideal period for intervention. We subsequently received CIHR bridge funds to conduct a pilot study examining Tele-CBT 1 year post-surgery. Patients (*N* = 21) in this study similarly reported improvements in eating pathology and depression, as well as high level of treatment satisfaction. Collectively, our pilot studies suggest that recruitment is achievable, delivery of CBT by telephone is feasible, patients report a high level of treatment satisfaction and improvements in eating pathology and psychological distress immediately following the intervention, and patients and clinicians recommend that CBT be delivered approximately 1 year following surgery. Thus, we have laid the foundation for our proposed RCT, which will examine whether Tele-CBT delivered 1 year post-surgery is efficacious in optimizing weight loss and improving physical and psychological functioning up to 3 years post-surgery.

Purpose of Study:

The **primary objective** of this 2-arm RCT is to examine the efficacy of Tele-CBT (7 sessions delivered 12 months following surgery) as an adjunctive treatment to the usual standard of bariatric care in optimizing weight loss and improving medical burden, eating pathology, psychological distress, and quality of life. Participants identified as meeting the study inclusion/exclusion criteria will be randomly assigned to either: 1) Treatment-As-Usual (TAU) Control group (i.e., bariatric surgery + routine clinic visits), or 2) Tele-CBT (i.e., bariatric

surgery + routine clinic vists + post-operative Tele-CBT).

The **secondary objective** is to examine improvements in medical burden, eating pathology, psychological distress, and quality of life.

Outcome Measures:

The primary outcome will be weight measured in kilograms. The secondary outcomes measures will be obesity related comorbidities and changes in eating pathology, as well as changes in depression, anxiety and quality of life. See measures in Appendix.

Hypotheses:

1. Tele-CBT will lead to significantly lower weight 2 and 3 years following surgery compared to standard care.
2. Tele-CBT will lead to alleviation of medical burden and improvements in eating pathology, psychological distress, and quality of life extending to 3 years following surgery compared to standard care.

Methods

Participants:

Recruitment during our pilot studies suggests that 350 participants (and 248 completers) is a feasible recruitment goal over this time period, with 200 participants to be recruited from Toronto Western Hospital and 150 participants to be recruited from Humber River Hospital. We have completed several pilot studies on Tele-CBT, thus, our research infrastructure is already set up. We plan to recruit 144 participants per year for the first 2.5 years of the study. Recruitment will end in the latter half of year 3, and primary and secondary outcomes will be assessed for the final 2 years of the study. To be eligible for bariatric surgery at Toronto Western Hospital, patients must be over the age of 18 and have a body mass index of 35 kg/m^2 or greater. All bariatric surgery candidates who meet the study inclusion/exclusion criteria will be eligible for participation

Inclusion criteria:
1. Age 18 to 65 years
2. Received bariatric surgery 1 year ago
3. Fluent in English
4. Have Internet access to complete online questionnaires

Exclusion criteria:
1. Current active suicidal ideation
2. Current poorly controlled psychiatric illness that would render Tele-CBT very difficult, including serious mental illness (i.e., psychotic disorder, bipolar disorder), severe depression (i.e., current major depressive episode diagnosis and Patient Health

Questionnaire [PHQ-9][58] score > 20), or severe anxiety (i.e., current anxiety disorder diagnosis and Generalized Anxiety Disorder [GAD-7][59] score >15);
3. Current poorly controlled medical illness that would render Tele-CBT very difficult.

Of note, patients in the Bariatric Surgery Program who have active suicidal ideation, serious mental illness, severe depression, or severe anxiety do not receive bariatric surgery until their psychiatric symptoms stabilize. Thus, the vast majority patients who are currently considered appropriate candidates for bariatric surgery will be eligible for participation in this study. In the event that participants develop significant mental health issues (e.g., active suicidal ideation, serious mental illness) during the research study, a consultation will be arranged with one of the staff psychiatrists in the program.

Sample Size and Statistical Analysis:

The study employs a 2-arm RCT design. Participants' weights will be measured at 5 time points (1, 1.25, 1.5, 2, and 3 years post-surgery). The primary goal is to determine if the mean weights differ between the Tele-CBT and TAU Control groups at 2 years post-surgery. In previous research on the impact of CBT programs, weight loss is typically in the range of 7.5% to 10%.[31,32] Discussion with bariatric surgeons has indicated that a difference of 5% in weight loss would be impressive enough to warrant a program to implement CBT. In our clinic data, we find that the mean weight at 2 years postoperation is 92kg, the correlation between 1 and 2 year weights is 0.8, and the between-subject standard deviation at 1 year and 2-years is 21kg. A 5% difference in weight at 2 years (equivalently, a difference in 1 to 2 year weight change equal to 5% of the 2-year weight) is a clinically important difference and translates to approximately 4.5kg. With a type I error rate of 5%, if the true difference in weights between the Control and Tele-CBT groups at 2 years is 4.5kg, a sample size of 124 per group gives 80% power in an analysis of covariance with 1 year weight as the covariate. Anticipating up to 30% loss to follow-up or withdrawal between 1 and 2 years, we will enroll 175 participants per group.

Each year, the TWH-BSP reassesses 336 patients at 1 year post-op. We plan to recruit for 2.5 years; thus, as many as 840 patients will be approached to participate over the recruitment period. Based on our pilot studies, we estimate that 50% of participants approached will be eligible to participate and interested in receiving CBT (as many as 168 per year; 420 across 2.5 years). In order to ensure 248 study completers, we will recruit 350 participants to conservatively account for up to 30% of participants withdrawing during treatment or lost to follow-up.

The **primary analysis** will compare mean weights in the Tele-CBT and TAU Control groups at 2 years post-surgery using analysis of covariance, with 1-year post-surgery weight and stratification variables sex and recruitment site as the covariates. We will investigate individual patient weight trajectories over time (1 year [pre-CBT], 1.25 years [post-CBT], 1.5 years, 2 years, and 3 years postsurgery). We will plot these trajectories, investigating patterns in each of the groups. Additionally, we will use linear mixed effects models to assess whether patterns of changes over the entire 3-year postsurgery period differ in the Tele-CBT and TAU Control groups while handling the longitudinal and correlated nature of the data.

The **secondary hypotheses** are concerned with comparing indicators of medication pill burden and remission of obesity-related comorbidities as per clinical records. Given the durable benefit of bariatric surgery on metabolic indices such as T2DM,[20] we will specifically compare

complete remission (i.e. A1C<6.0%, fasting plasma glucose (FPG)<6.0 mmol/L, and no hypoglycemic medications) in the Tele-CBT and Standard Care Control groups at 2 and 3 years post-surgery. Participants will be divided into 3 groups: (1) T2DM, (2) T2DM in partial remission (i.e. A1C=6.0-6.4%, fasting plasma glucose (FPG)=6.1-6.9 mmol/L, and no hypoglycemic medications), and (3) T2DM in complete remission. We will compare the proportions in these three groups between the Tele-CBT and Control groups using a chi-squared test on the corresponding 2x3 contingency table. We will also compare Tele-CBT and Control groups on the proportions meeting criteria for T2DM remission. The secondary hypotheses are also concerned with comparing eating pathology, psychological distress, and quality of life measures across the Tele-CBT and Control groups, and the approach for longitudinal analysis of the primary outcome will also be used to compare these groups. Despite evidence of sex differences among bariatric surgery patients,[22] they have been largely ignored in research on psychosocial interventions. Males comprise only 20% of all patients enrolled in bariatric surgery programs, a percentage that is not reflective of the sex distribution of extreme obesity. We will carry out a subgroup analysis to examine whether the efficacy of Tele-CBT differs between the sexes. We will also assess sex differences in the percentage of individuals who decline participation in the study, drop out from treatment, or are lost to follow-up.

Procedure:

The proposed 2-arm RCT will examine the efficacy of Tele-CBT delivered 1 year post-surgery as an adjunctive treatment to standard bariatric care in a real-world clinical setting. A total of 350 bariatric surgery patients will be recruited from the Bariatric Surgery Programs at Toronto Western Hospital (TWH-BSP) and Humber River Hospital (HRH-BSP) and be randomized to either: 1) Treatment as Usual (i.e., bariatric surgery + routine clinic visits), or 2) Tele-CBT (i.e., bariatric surgery + routine clinic visits + post-operative Tele-CBT). See Figure 1 for Study Design.

Figure 1: Study Design

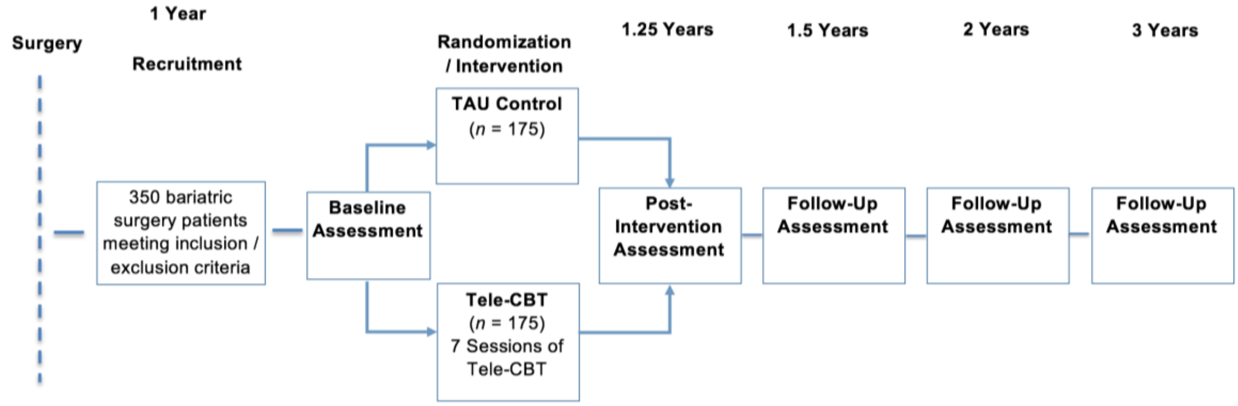

Treatment as Usual (Standard Bariatric Care Control): Participants assigned to the Standard Bariatric Care group will attend routine clinic visits at the TWH-BSP. These visits generally include education on bariatric surgery and nutrition. Patients meet with select members

of the multidisciplinary team at 1 year post surgery with physician follow-up at 2 and 3 year post-surgery, and may attend an optional monthly support group. Participants' service utilization (i.e., attendance at optional sessions) will be documented and compared across groups. Participants will also be asked to weigh themselves during the assessment points that do not correspond to clinic visits (i.e., 1.25 and 1.5 years post-surgery) and to take a picture of the weight on the scale to send via email to the study coordinator to increase the reliability of self-report.

Post-Op Telephone-Based CBT: The Post-Op Tele-CBT intervention will be delivered one year following bariatric surgery. According to longitudinal research, as well as our own clinical data and pilot studies, patients typically experience rapid weight loss during the first 6 months following surgery with little effort,[55,56] and during this "honeymoon period", have little incentive to engage in psychosocial interventions. In addition, 1 year post-op was selected because patients are typically medically stable at this point. In addition, they have not yet entered the high-risk period for weight regain that typically occurs 1.5 to 2 years following surgery,[55,56] but they do have to put more effort into losing weight relative to the first 6 months. Participants will receive 6 weekly Tele-CBT sessions and 1 final "booster" session 1 month later, all approximately 55-minutes in duration and scheduled at a time convenient for the participants. The development of the Tele-CBT protocol and the content of the sessions, as well as preliminary evidence for the feasibility, acceptability and efficacy of the protocol, have been previously described by our team.[52-54] Briefly, the Tele-CBT sessions focus on introducing the cognitive behavioural model of overeating and obesity, scheduling healthy meals and snacks at regular time intervals and recording consumption using food records, scheduling pleasurable alternative activities to overeating, identifying and planning for difficult eating scenarios, and reducing vulnerability to overeating by solving problems and challenging negative thoughts. Participants are expected to complete CBT homework between sessions, such as completing food records, engaging in pleasurable and self-care activities, and completing a variety of worksheets. Three clinical psychology graduate students and psychiatry residents/fellows will work as study therapists under the supervision of Drs. Cassin and Sockalingam, respectively. All therapists will receive training in the Tele-CBT protocol and will have biweekly case supervision meetings. Participants in this group will also be asked to weigh themselves during the assessment points that do not correspond to clinic visits (i.e., 1.25 and 1.5 years post-surgery) and to take a picture of the weight on the scale to send via email to the study coordinator to increase the reliability of self-report.

Participants will be recruited using the same practices that have proven very effective in our Tele-CBT pilot studies at the TWH-BSP. Specifically, when clinic staff contact patients by telephone to remind them of their routine clinic visit scheduled for 1, 3, or 12 months post-surgery, they will ask patients if they are willing to be contacted by a member of the research team regarding the Tele-CBT study. The research coordinator will subsequently contact those patients by telephone or e-mail (if the participant consents) to explain the study in greater detail. Those interested in participating will be provided with the consent form in person at their appointment to review and sign. Patients who are interested in participating in the study but cannot come in person to sign the consent form (i.e., those who complete their appointments via Telehealth Ontario) will be mailed a copy of the consent form with a prepaid return envelope to review, sign, and mail back. Once patients have provided their consent, they will be screened for the inclusion/exclusion criteria noted below, and will undergo a diagnostic interview (the MINI International Neuropsychiatric Inventory[57]) to assess for exclusionary mental disorders. Once

screening is complete, eligible and consenting participants will be e-mailed a link to the online baseline questionnaire packet administered through *Qualtrics* survey software and they will be weighed when they return to the TWH-BSP for their 12-month post-operative routine clinic visit. Upon completion of these baseline measures, the research coordinator will randomly assign participants to either the Tele-CBT group or Control group. Randomization will occur via an independent central web-based system to ensure allocation concealment with stratification by sex, given the documented sex differences in bariatric surgery patients.[22] The study biostatistician will generate the 1:1 randomization sequence with a computerized random number generator, using a random permuted block design with randomly chosen block sizes.

Measures (See Appendix):

Self-Report Questionnaires

Patient Health Questionnaire (*PHQ-9*)[58] – The PHQ-9 is a 9-item self-report measure of depression severity. Respondents are asked to rate the frequency with which they have experienced depressive symptoms over the last two weeks on a scale ranging from 0 (not at all) to 3 (nearly every day). Scores on the PHQ-9 can range from 0 to 27, and mild, moderate, moderately severe, and severe levels of depressive symptoms correspond to cut-off scores of 5, 10, 15, and 20 respectively.

Generalized Anxiety Disorder Questionnaire (*GAD-7*)[59] – The GAD-7 is a 7-item self report measure of anxiety severity. It was originally developed to diagnose generalized anxiety disorder, but it has also proved to be a good screening instrument for other disorders including panic disorder, social phobia, and post-traumatic stress disorder. Respondents are asked to rate the frequency with which they have experienced anxiety symptoms over the last two weeks on a scale ranging from 0 (not at all) to 3 (nearly every day). Scores on the GAD-7 can range from 0 to 21, and mild, moderate, and severe levels of anxiety symptoms correspond to cut-off scores on 5, 10, and 15 respectively.

Binge Eating Scale (*BES*)[60] – The BES is a 16-item self-report measure that assesses the presence of binge eating behaviour indicative of an eating disorder. It was devised specifically for use with obese individuals (68). Scores on the BES range from 0 to 46, and moderate and severe levels of binge eating correspond to cut-off scores of 18 and 27.

Loss of Control Over Eating Scale (LOCES-Brief)[61] – the LOCES-Brief is a 7-item self-report measure that assesses the behavioural and cognitive aspects of loss of control eating, which may occur even in the absence of objectively large eating binges.

Emotional Eating Scale (*EES*)[62] – The EES is a 25-item self-report measure that assesses the tendency to cope with negative affect by eating. Respondents are presented with 25 emotions and are asked to rate the strength of their urge to eat on a scale from 1 (no desire to eat) to 5 (an overwhelming urge to eat) when experiencing each of the emotions. The EES consists of 3 subscales reflecting anger/frustration, anxiety, and depression.

EuroQol (*EQ-5D-5L*)[63] – The EQ-5D-5L is a 25 item self-report measure of health-related quality of life. The EQ-5D-5L coverts the domains of mobility, self-care, daily activities, pain/discomfort, and anxiety/depression.

Measures of Medical Burden

Assessed at routine clinic visits at 1 year (Pre-Intervention), 2 years, and 3 years following surgery. Bloodwork on obesity related medical comorbidities are collected as part of routine clinical follow-up after bariatric surgery and will be accessed through patients' medical records.

- *Overall medication pill burden* (assessed by physician discontinuation)
- *Diabetes specific medication burden* (assessed by physician discontinuation)
- *Sustained remission of obesity related comorbidities* (e.g. hypertension; assessed by laboratory values)
- *Sustained remission of Type 2 Diabetes mellitus* (assessed by hemoglobin A1C and fasting plasma glucose levels)

Privacy and Confidentiality:

E-mail correspondence will be sent from a secure UHN e-mail account. Participants will be notified that security and confidentiality of information cannot be guaranteed through e-mail correspondence. All rating scales and forms used at each assessment will use a single coding system, with only the research ID number and study name written on it. Personal health information is required for determining participant eligibility for the study and to contact the participant during the study for Tele-CBT sessions. The master code will be kept by the research coordinator for the duration of the study and will be kept separately from the research data (i.e., participant files). Contact information will be used throughout the course of the study and correspond to the master code. All data and personal health information will be stored in a locked room in a locked cabinet. Contact information, demographic data, and computerized rating scales will be stored on a password-protected computer on a secure network drive. A login name and password will be required to access these files and the computer will be located in a locked office.

Data Transfer Between Sites:

Select data will be transferred from UHN to Ryerson, as listed below. The Tele-CBT therapists will obtain the contact information of participants from the research coordinator via telephone so they can contact them for Tele-CBT sessions. The research coordinator will only provide the participant's name, phone number and email and no other identifying information or personal health information. The session notes that the therapists write following the Tele-CBT sessions will be stored in a locked filing cabinet in the secure HEAL lab at Ryerson and only students working in the HEAL lab as therapists under the supervision of co-PI Dr. Stephanie Cassin will have access to this information. These notes will only indicate the participant ID and will not have any patient identifying information. The research coordinator will use the CRR (clinical research record) function on EPR to indicate on the patient's electronic medical record that they are currently enrolled in the UHN-based research study.

All of the Tele-CBT sessions will be audio-recorded with the use of a password protected digital audio recorder. All audio-recorded Tele-CBT sessions will be password protected and will remain at Ryerson until grading for adherence to protocol has been completed, at which time they will be deleted. Tele-CBT audio recordings will not be transferred back and forth between sites.

No data will be collected from Humber River Hospital. We will be consenting patients from both UHN and Humber River Hospital, and Humber will be helping to identify patients eligible for the study. As the Tele-CBT intervention is provided remotely, the process will remain the same for all UHN and Humber River Hospital consented patients. Data collected from Humber River Hospital patients will be stored with UHN patient data at UHN on a password-protected computer or in a locked cabinet within a locked room that only team members can access. Ryerson's role for Humber River Hospital patients, like UHN patients, are as mentioned above.

Risks and Benefits:

Risks:

Participants will be asked to reflect upon some personal issues and their psychological health (e.g., eating habits, mood, anxiety, quality of life) during Cognitive Behavioural Therapy and while completing the questionnaires. Participants may choose to discontinue Cognitive Behavioural Therapy or to refuse to answer questions at any time if they experience discomfort.

Benefits:

Our pilot studies suggest that the Tele-CBT intervention improves binge eating, emotional eating, depression, and anxiety.[65,66] In addition, previous randomized controlled trials in non-bariatric surgery populations have demonstrated that CBT is efficacious in improving binge eating[66], depression[67], and anxiety[67]. Thus, we believe that patients have the potential to benefit as a result of taking part in this study.

Implications:

If Tele-CBT is found to be efficacious, it could potentially become the standard of care for bariatric surgery patients, including those who do not live within driving distance of bariatric centres or who cannot attend weekly treatment sessions due to practical barriers. Given the minimal exclusion criteria for this study, most bariatric surgery patients will be eligible to participate, making the results generalizable to other settings beyond the study recruitment site. The manualized telephone-based protocol is associated with a low intervention burden for health care systems and patients, and is thus highly feasible for implementation elsewhere. The identification of efficacious psychosocial interventions will become increasingly important as the prevalence of extreme obesity and obesity-related medical comorbidities continue to rise, and as the number of bariatric surgeries performed each year increases.

Conflicts of Interest:

There are no known conflicts of interest.

Study Budget:

This study is being funded by the Canadian Institutes for Health Research (Project grant valued at $489,600).

References

1. Di Ruggiero, E., Frank, J., & Moloughney, B. (2004). Strengthen Canada's public health system now. *Canadian Journal of Public Health, 95*, 5-11.

2. Lau, D. C. W., Douketis, J. D., Morrison, K. M., Hramiak, I. M., Sharma, A. M., & Ur, E. (2007). 2006 Canadian clinical practice guidelines on the management and prevention of obesity in adults and children. *Canadian Medical Association Journal, 176* (8 Suppl), S1-S13.

3. Stewart, S. T., Cutler, D. M., & Rosen, A. B. (2009). Forecasting the effects of obesity and smoking on U.S. life expectancy. *New England Journal of Medicine, 361*, 2252-2260.

4. World Health Organization (2000). *Obesity: Preventing and managing the global epidemic.* WHO Technical Report Series 894.

5. Shields, M., Carroll, M. D., & Ogden, C. L. (2011). Adult obesity prevalence in Canada and the United States. *NCSH Data Brief*, number 56.Hyattsville, MD: National Centre for Health Statistics.

6. Flegal, K.M., Carroll, M.D., Ogden, C.L., & Johnson, C.L. (2002). Prevalence and trends in obesity among US adults, 1999-2000. *Journal of the American Medical Association, 288,* 1723-1727.

7. Sturm, R. (2003). Increases in clinically severe obesity in the United States, 1986-2000. *Archives of Internal Medicine, 163,* 2146-2148.

8. Bray, G. (2004). Medical consequences of obesity. *The Journal of Clinical Endocrinology and Metabolism, 89*, 2583–2589.

9. Guh, D. P., Zhang, W., Bansback, N., Amarsi, Z., Birmingham, L., & Anis, A. H. (2009). The incidence of comorbidities related to obesity and overweight: A systemtic review and metaanalysis. *Biomed Central (BMC) Public Health, 9*, 88-108.

10. Padwal, R. S., Pajewski, N. M., Allison, D. B., & Sharma, A. M. (2011). Using the Edmonton obesity staging system to predict mortality in a population-representative cohort of people with overweight and obesity. *Canadian Medical Association Journal, 183*, E1059-1066.

11. National Institute for Health and Clinical Excellence (2006). *Obesity: Guidance on the prevention, identification, assessment, and management of overweight and obesity in adults and children* (NICE clinical guideline 43). London: National Institute for Health and Clinical Excellence.

12. Buchwald, H., & Williams, S. E. (2004). Bariatric surgery worldwide 2003. *Obesity Surgery, 14,* 1157-1164.

13. Samuel, I., Mason, E., Renquist, K., Huang, Y., Zimmerman, M., & Jamal, M. (2006). Bariatric surgery trends: An 18-year report from the International Bariatric Surgery Registry. *American Journal of Surgery, 192*, 657-662.

14. Mechanick, J. I., Kushner, R. F., Sugarman, H. J., Gonzales-Campoy, J. M., Collazo-Clavell, M. L.,Spitz, A. F., et al. (2009). American Association of Clinical Endocrinologists, The Obesity Society,and American Society for Metabolic and Bariatric Surgery medical guidelines for clinical practice for the perioperative nutritional, metabolic, and non-surgical support of the bariatric surgery patient. *Obesity, 17 (Suppl1)*, S1-S70.

15. Tadross, J. A., & le Roux, C. W. (2009). The mechanisms of weight loss after bariatric surgery. *International Journal of Obesity, 33*, S28-S32.

16. Adams, T. D., Pendleton, R. C., Strong, M. G., Kolotkin, R. L., Walker, J. M., Litwin, S. E., et al. (2010). Health outcomes of gastric bypass patients compared to nonsurgical nonintervened severely obese. *Obesity, 18*, 121-130.

17. Sjostrom, L., Narbo, K., Sjostrom, C. D., Karason, K., Larsson, B., Wedel, H., et al. (2007). Effects of bariatric surgery on mortality in Swedish obese subjects. *New England Journal of Medicine, 357*, 741-752.

18. Buchwald, H., Avidor, Y., Braunwald, E., Jensen, M. D., Pories, W., Fahrbach, K., et al. (2004). Bariatric surgery: a systematic review and meta-analysis. *Journal of the American Medical Association, 292*, 1724–1737.

19. Shah, M., Simha, V., & Garg, A. (2006). Long term impact of bariatric surgery on body weight, comorbidities and nutritional status. *The Journal of Clinical Endocrinology & Metabolism, 91*,4223–4231.

20. Courcoulas, A. P., Christian, N. J., Belle, S. H., Berk, P. D., Flum, D. R., Garcia, L., et al. (2013). Weight change and health outcomes at 3 years after bariatric surgery among individuals with severe obesity. *Journal of the American Medical Association, 310*, 2416-2425.

21. Magro, D. O., Geloneze, B., Delfini, R., Pereja, B. C., Callejas, F., & Pereja, J. C. (2008). Longterm weight regain after gastric bypass: A 5-year prospective study. *Obesity Surgery,18*, 648-651.

22. DiGiorgi, M., Rosen, D. J., Choi, J. J., Milone, L., Schrope, B., Olivero-Rivera, L. et al. (2010). Re-emergence of diabetes after gastric bypass in patients with mid- to long-term follow-up. *Surgery for Obesity and Related Diseases, 6*, 249-253.

23. Shah, M., Simha, V., & Garg, A. (2006). Long term impact of bariatric surgery on body weight, comorbidities and nutritional status. *The Journal of Clinical Endocrinology & Metabolism, 91*, 4223–4231.

24. Meany, G., Conceicao, E., & Mitchell, J. E. (2014). Binge eating, binge eating disorder, and loss of control eating: Effects on weight outcomes after bariatric surgery. *European Eating Disorders Review, 22*, 87-91.

25. Sheets, C., Peat, C., Berg, K., White, E., Bocchieri-Ricciardi, L., Chen, E., & Mitchell, J. E. (2015). Post-operative psychosocial predictors of outcome in bariatric surgery. *Obesity Surgery, 25*, 330-345.

26. Muhlhans, B., Horbach, T., De Zwaan, M. (2009). Psychiatric disorders in bariatric surgery candidates. A review of the literature and results of a German prebariatric surgery sample. *General Hospital Psychiatry, 31*, 414-421.

27. Kalarchian, M. A., & Marcus, M. D. (2015). Psychosocial interventions pre and post bariatric surgery. *European Eating Disorders Review, 23*, 457-462.

28. Beck, N. N., Johannsen, M., Stoving, R. K., Mehlsen, M., & Zachariae, R. (2012). Do postoperative psychotherapeutic interventions and support groups influence weight loss following bariatric surgery? A systematic review and meta-analysis of randomized and nonrandomized trials. *Obesity Surgery, 22*, 1790-1797.

29. National Institute for Health and Care Excellence. Depression in adults: Recognition and management. NICE clinical guideline 90. 2009. http://www.nice.org.uk/guidance/cg90.

30. National Institute for Health and Care Excellence. *Eating disorders in over 8's: Management*. NICE clinical guideline 9. 2004. http://www.nice.org.uk/guidance/cg9.

31. Cooper, Z., Doll, H. A., Hawker, D. M., Bryne, S., Bonner, G., Eeley, E., et al. (2010). Testing a new cognitive behavioral treatment for obesity: A randomized controlled trial with three year follow-up. *Behavior Research and Therapy, 48*, 706-713.

32. Werrij, M. Q., Jansen, A., Mulkens, S., Elgersma, H. J., Ament, A., Hospers, H. J. (2009). Adding cognitive therapy to dietetic treatment is associated with less relapse in obesity. *Journal of Psychosomatic Research, 67*, 315-324.

33. Cassin, S. E., & Atwood, M. E. (in press). Cognitive behavioural therapy for severe obesity. In S Sockalingam & R. Hawa (Eds.), *Psychiatric care in severe obesity: An interdisciplinary guide to integrated care*. New York, NY: Springer.

34. Atwood, M. E., David, L., & Cassin, S. E. (in press). Cognitive behavioural therapy for bariatric surgery patients. In R. Rajendram, C. Martin, & V. R. Preedy (Eds.), *Pathophysiology of bariatric surgery: Metabolism, nutrition, procedures, outcomes and adverse effects.* London, UK: Academic Press.

35. Kalarchian, M. A., Marcus, M. D., Courcoulas, A. P., Cheng, Y., Levine, M. D., & Josbeno, D. (2012). Optimizing long-term weight control after bariatric surgery: A pilot study. *Surgery for Obesity and Related Diseases, 8,* 710-715.

36. Gade, H., Friborg, O., Rosenvinge, J. H., Småstuen, M. C., & Hjelmesæth, J. (2015). The impact of a preoperative cognitive behavioural therapy (CBT) on dysfunctional eating behaviours, affective symptoms and body weight 1 year after bariatric surgery: A randomized controlled trial. *Obesity Surgery, 25,* 2112-2119.

37. King, W. C., Engel, S. G., Elder, K. A., et al. (2012). Walking capacity of bariatric surgery candidates. *Surgery for Obesity and Related Diseases, 8,* 48-59.

38. Lara, M. D., Baker, M. T., Larson, C. J., Mathiason, M. A., Lambert, P. J., & Kothari, S. N. (2005). Travel distance, age, and sex as factors in follow-up visit compliance in the post-gastric bypass population. *Surgery for Obesity and Related Diseases, 1*, 17-21.

39. Sockalingam, S., Cassin, S. E., Hawa, R., Khan, A., Wnuk, S., & Okrainec, A. (2013). Predictors of postbariatric surgery appointment attendance: The role of attachment style. *Obesity Surgery (*Epub ahead of print).

40. Lara, M. D., Baker, M. T., Larson, C. J., Mathiason, M. A., Lambert, P. J., & Kothari, S. N. (2005). Travel distance, age, and sex as factors in follow-up visit compliance in the post-gastric bypass population. *Surgery for Obesity and Related Diseases, 1*, 17-21.

41. Moroshko, I., Brennan, L., & O'Brien, P. (2012). Predictors of attrition in bariatric aftercare: A systematic review of the literature. *Obesity Surgery* (online first).

42. Hart. S. L., & Hart, T. A. (2010). The future of cognitive behavioural interventions within behavioural medicine. *Journal of Cognitive Psychotherapy, 24*, 344-353.

43. Eakin, E. G., Lawler, S. P., Vandelanotte, C., & Owen, N. (2007). Telephone interventions for physical activity and dietary behaviour change: A systematic review. *American Journal of Preventative Medicine, 32*, 419-434.

44. Wells, A. M., Garvin, V., Dohm, F. A., & Striegel-Moore, R. H. (1997). Telephone based guided self-help for binge eating disorder: A feasibility study. *International Journal of Eating Disorders, 21*, 341-346.

45. Ludman, E. J., Simon, G. E., Tutty, S., & Von Korff, M. (2007). A randomized trial of telephone psychotherapy and pharmacotherapy for depression: Continuation and durability of effects. *Journal of Consulting and Clinical Psychology, 75*, 257-266.

46. Mohr, D. C., Hart, S., L., Julian, L., Catledge, C., Honos-Webb, L., Vella, L., & Tasch, E. T. (2005). Telephone-administered psychotherapy for depression. *Archives of General Psychiatry, 62*, 1007-1014.

47. Tutty, S., Sprangler, D. L., Poppleton, L. E., Ludman, E. J., & Simon, G. E. (2010). Evaluating the evidence of cognitive behavioural teletherapy in depressed adults. *Behaviour Therapy, 41*, 229-236.

48. Tutty, S., Ludman, E. J., & Simon, G., (2005). Feasibility and acceptability of a telephone psychotherapy program for depressed adults treated in primary care. *General Hospital Psychiatry, 27*, 400-410.

49. Mohr, D. C., Ho, J., Duffecy, J., Reifler, D., Sokol, L., Burns, M. N., et al. (2012). Effect of telephone-administered versus face-to-face cognitive behavioural therapy on adherence to therapy and depression outcomes among primary care patients. *Journal of the American Medical Association, 307*, 2278-2285.

50. Young, J. & Beck, A. T. (1980). *The Cognitive Therapy Scale rating manual*. Bala Cynwyd, MD: Beck *Institute* for Cognitive Behaviour Therapy.

51. Totten, A. M., Womack, D. M., Eden, K. B., McDonagh, M. S., Griffin, J. C., Grusing, S., Hersh, W. R. Telehealth: Mapping the evidence for patient outcomes from systematic reviews. Technical Brief No. 26. (Prepared by the Pacific Northwest Evidence-based Practice Center under Contract No. 290-2015-00009-I.) AHRQ Publication No.16-EHC034-EF. Rockville, MD: Agency for Healthcare Research and Quality; June 2016. www.effectivehealthcare.ahrq.gov/reports/final.cfm.

52. Cassin, S. E., Sockalingam, S., Wnuk, S., Strimas, R., Royal, S., Hawa, R., & Parikh, S. V. (2013). Cognitive behavioural therapy for bariatric surgery patients: Preliminary evidence for feasibility, acceptability, and effectiveness. *Cognitive and Behavioural Practice, 20*, 529-543.

53. Cassin, S. E., Sockalingam, S., Du, C., Wnuk, S., Hawa, R., & Parikh, S. (2016). A pilot randomized controlled trial of telephone-based cognitive behavioural therapy for preoperative bariatric surgery patients. *Behaviour Research and Therapy, 80*, 17-22.

54. Sockalingam, S., Cassin, S. E., Wnuk, S., Du, C., Jackson, T., Hawa, R., & Parikh, S. (in press). A pilot study on telephone cognitive behavioural therapy for patients six months post-bariatric surgery. *Obesity Surgery*.

55. Karlsson, J., Sjostrom, L., & Sullivan, M. (1998). Swedish Obese Subjects (SOS) an intervention study of obesity. Two-year follow-up of health-related quality of life and eating behaviour after gastric surgery for extreme obesity. *International Journal of Obesity, 22*, 113-126.

56. Ryden, A., & Torgerson, J. S. (2006). The Swedish obese subjects study—What has been accomplished to date? *Surgery for Obesity and Related Diseases, 2*, 549-560.

57. Sheehan, D. V., Lecrubier, Y., Sheehan, K. H., Amorim, P., Janavs, J., Weiller, E., Herqueta, T., Baker, R., & Dunbar, G. C. (1998). The Mini-International Neuropsychiatric Interview for DSMIV and ICD-10. *Journal of Clinical Psychiatry, 59* (Suppl 20), 22-33.

58. Spitzer, R. L., Kroenke, K., Williams, J. B., & The Patient Health Questionnaire Primary Care Study Group. (1999). Validation and utility of a self-report version of PRIME-MD: the PHQ primary care study. *JAMA: The Journal of the American Medical Association, 282*, 1737-1744.

59. Spitzer, R. L., Kroenke, K., Williams, J. B. W., & Lowe, B. (2006). A brief measure for assessing generalized anxiety disorder – the GAD-7. *Archives of Internal Medicine, 166*, 1092-1097.

60. Gormally, J., Black, S., Daston, S., & Rardin, D. (1982). The assessment of binge eating severity among obese persons. *Addictive Behaviors, 7*, 47–55.

61. Latner, J., Mond, J. M., Kelly, M. C., Haynes, S. N., & Hay, P. J. (2014). The loss of control over eating scale: Development and psychometric evaluation. *International Journal of Eating Disorders, 47*, 647-659.

62. Arnow, B., Kenardy, J., & Agras, W. S. (1995). The Emotional Eating Scale: The development of a measure to assess coping with negative affect by eating. *International Journal of Eatin Disorders, 18*, 79-90.

63. EuroQol Group, EuroQol (1990). A new facility for the measurement of health-related quality of life. *Health Policy, 16*, 199-208.

64. Cassin, S. E., Sockalingam, S., Du, C., Wnuk, S., Hawa, R., & Parikh, S. (2016). A pilot randomized controlled trial of telephone-based cognitive behavioural therapy for preoperative bariatric surgery patients. *Behaviour Research and Therapy, 80*, 17-22.

65. Sockalingam, S., Cassin, S. E., Wnuk, S., Du, C., Jackson, T., Hawa, R., & Parikh, S. (2017). A pilot study on telephone cognitive behavioural therapy for patients six months post-bariatric surgery. *Obesity Surgery, 27*, 670-675.

66. Brownley, K. A., Berkman, N. D., Sedway, J., A., Lohr, K. N., & Bulik, C. M. (2007). Binge eating disorder treatment: A systematic review of randomized controlled trials. *International Journal of Eating Disorders, 40*, 337-348.

67. Tolin, D. F. (2010). Is cognitive behavioural therapy more effective than other therapies? A meta-analytic review. *Clinical Psychology Review, 30*, 710-720.

Appendix: **Measures**

Patient Health Questionnaire or PHQ-9

Over the last 2 weeks, how often have you been bothered by any of the following problems? Please circle the appropriate number.

	**Not at all**	**Several days**	**More than half the days**	**Nearly every day**
1. Little interest or pleasure in doing things	0	1	2	3
2. Feeling down, depressed, or hopeless	0	1	2	3
3. Trouble falling or staying asleep, or sleeping too much	0	1	2	3
4. Feeling tired or having little energy	0	1	2	3
5. Poor appetite or overeating	0	1	2	3
6. Feeling bad about yourself – or that you are a failure or have let yourself or your family down	0	1	2	3
7. Trouble concentrating on things, such as reading the newspaper or watching television	0	1	2	3
8. Moving or speaking so slowly that other people could have noticed. Or the opposite – being so fidgety or restless that you have been moving around a lot more than usual	0	1	2	3
9. Thoughts that you would be better off dead or of hurting yourself in some way	0	1	2	3
(For staff coding: Total Score _____	=	_____ +	_____ +	_____ **)**
PHQ 9	=	_____	**Reviewed by:**	_____

If you check off any of these problems, how difficult have these problems made it for you to do your work, take care of things at home, or get along with other people?

Not at all difficult	Somewhat difficult	Very difficult	Extremely difficult
€	€	€	€

Generalized Anxiety Disorder 7 or GAD-7

Over the last 2 weeks, how often have you been bothered by any of the following problems? Please circle the appropriate number.

	Not at all	Several days	More than half the days	Nearly every day
1. Feeling nervous, anxious or on edge	0	1	2	3
2. Not being able to stop or control worrying	0	1	2	3
3. Worrying too much about different things	0	1	2	3
4. Trouble relaxing	0	1	2	3
5. Being so restless that it is hard to sit still	0	1	2	3
6. Becoming easily annoyed or irritable	0	1	2	3
7. Feeling afraid as if something awful might happen	0	1	2	3

Loss of Control Over Eating Scale or LOCES

In the **past month**, how often have you had the following experiences during a time when you were eating? Please respond to each item using the following scale:

1	2	3	4	5
Never	Rarely	Occasionally	Often	Always

IN THE PAST MONTH:	Never	Rarely	Occasionally	Often	Always
1. I continued to eat past the point when I wanted to stop.	1	2	3	4	5
2. I felt like I had “blown it” and might as well keep eating.	1	2	3	4	5
3. I felt helpless about controlling my eating.	1	2	3	4	5
4. My eating felt like a ball rolling down a hill that just kept going and going.	1	2	3	4	5
5. I found myself eating despite negative consequences.	1	2	3	4	5
6. I felt like the craving to eat overpowered me.	1	2	3	4	5
7. I felt like I could not do anything other than eat.	1	2	3	4	5

Binge Eating Scale or BES

Below are groups of numbered statements. Read all of the statements in each group and **circle the one** that best describes the way you feel about your eating behavior.

1.

1. I don’t feel self-conscious about my weight or body size when I’m with others.
2. I feel concerned about how I look to others, but it normally does not make me feel disappointed with myself.
3. I do get self-conscious about my appearance and weight which makes me feel disappointed in myself.
4. I feel very self-conscious about my weight and frequently, I feel intense shame and disgust for myself. I try to avoid social contacts because of my self-consciousness.

2.

1. I don’t have any difficulty eating slowly in the proper manner.
2. Although I seem to “gobble down” foods, I don’t end up feeling stuffed because of eating too much.
3. At times, I tend to eat quickly and then, I feel uncomfortably full afterwards.
4. I have the habit of bolting down my food, without really chewing it. When this happens I usually feel uncomfortably stuffed because I’ve eaten too much.

3.

1. I feel capable to control my eating urges when I want to.
2. I feel like I have failed to control my eating more than the average person.
3. I feel utterly helpless when it comes to feeling in control of my eating urges.
4. Because I feel so helpless about controlling my eating I have become very desperate about trying to get in control.

4.

1. I don’t have the habit of eating when I’m bored.
2. I sometimes eat when I’m bored, but often I’m able to “get busy” and get my mind off food.
3. I have a regular habit of eating when I’m bored, but occasionally, I can use some other activity to get my mind off eating.
4. I have a strong habit of eating when I’m bored. Nothing seems to help me break the habit.

5.

1. I’m usually physically hungry when I eat something.
2. Occasionally, I eat something on impulse even though I really am not hungry.
3. I have the regular habit of eating foods, that I might not really enjoy, to satisfy a hungry feeling even though physically, I don’t need the food.
4. Even though I’m not physically hungry, 1 get a hungry feeling in my mouth that only seems to be satisfied when I eat a food, like a sandwich, that fills my mouth. Sometimes, when I eat the food to satisfy my mouth hunger, I then spit the food out so I won’t gain weight.

6.

1. I don't feel any guilt or self-hate after I overeat.
2. After I overeat, occasionally I feel guilt or self-hate.
3. Almost all the time I experience strong guilt or self-hate after I overeat.
4. I almost always feel a strong sense of guilt or regret after I overeat.

7.

1. I don't lose total control of my eating when dieting even after periods when I overeat.
2. Sometimes when I eat a "forbidden food" on a diet, I feel like I "blew it" and eat even more.
3. Frequently, I have the habit of saying to myself, "I've blown it now, why not go all the way" when I overeat on a diet. When that happens I eat even more.
4. I have a regular habit of starting strict diets for myself, but I break the diets by going on an eating binge. My life seems to be either a "feast" or "famine."

8.

1. I rarely eat so much food that I feel uncomfortably stuffed afterwards.
2. Usually about once a month, I eat such a quantity of food, I end up feeling very stuffed.
3. I have regular periods during the month when I eat large amounts of food, either at mealtime or at snacks.
4. I eat so much food that I regularly feel quite uncomfortable after eating and sometimes a bit nauseous.

9.

1. My level of calorie intake does not go up very high or go down very low on a regular basis.
2. Sometimes after I overeat, I will try to reduce my caloric intake to almost nothing to compensate for the excess calories I've eaten.
3. I have a regular habit of overeating during the night. It seems that my routine is not to be hungry in the morning but overeat in the evening.
4. In my adult years, I have had week-long periods where I practically starve myself. This follows periods when I overeat. It seems I live a life of either "feast or famine."

10.

1. I usually am able to stop eating when I want to. I know when "enough is enough."
2. Every so often, I experience a compulsion to eat which I can't seem to control.
3. Frequently, I experience strong urges to eat which I seem unable to control, but at other times I can control my eating urges.
4. I feel incapable of controlling urges to eat. I have a fear of not being able to stop eating voluntarily.

11.

1. I don’t have any problem stopping eating when I feel full.
2. I usually can stop eating when I feel full but occasionally overeat leaving me feeling uncomfortably stuffed.
3. I have a problem stopping eating once I start and usually I feel uncomfortably stuffed after I eat a meal.
4. Because I have a problem not being able to stop eating when I want, I sometimes have to induce vomiting to relieve my stuffed feeling.

12.

1. I seem to eat just as much when I’m with others (family, social gatherings) as when I’m by myself.
2. Sometimes, when I’m with other persons, I don’t eat as much as I want to eat because I’m self-conscious about my eating.
3. Frequently, I eat only a small amount of food when others are present, because I’m very embarrassed about my eating.
4. I feel so ashamed about overeating that I pick times to overeat when I know no one will see me. I feel like a “closet eater.”

13.

1. I eat three meals a day with only an occasional between meal snack.
2. I eat 3 meals a day, but I also normally snack between meals.
3. When I am snacking heavily, I get in the habit of skipping regular meals.
4. There are regular periods when I seem to be continually eating, with no planned meals.

14.

1. I don’t think much about trying to control unwanted eating urges.
2. At least some of the time, I feel my thoughts are pre-occupied with trying to control my eating urges.
3. I feel that frequently I spend much time thinking about how much I ate or about trying not to eat anymore.
4. It seems to me that most of my waking hours are pre-occupied by thoughts about eating or not eating. I feel like I’m constantly struggling not to eat.

15.

1. I don’t think about food a great deal.
2. I have strong cravings for food but they last only for brief periods of time.
3. I have days when I can’t seem to think about anything else but food.
4. Most of my days seem to be pre-occupied with thoughts about food. I feel like I live to eat.

16.

1. I usually know whether or not I’m physically hungry. I take the right portion of food to satisfy me.

2. Occasionally, I feel uncertain about knowing whether or not I'm physically hungry. At these times it's hard to know how much food I should take to satisfy me.
3. Even though I might know how many calories I should eat, I don't have any idea what is a "normal" amount of food for me.

<u>Emotional Eating Scale or EES</u>

We all respond to different emotions in different ways. Some types of feelings lead people to experience an urge to eat. Please indicate the extent to which the following feelings lead you to feel an urge to eat by <u>checking</u> the appropriate box.

		No Desire to Eat	A Small Desire to Eat	A Moderate Desire to Eat	A Strong Urge to Eat	An Overwhelming Urge to Eat
1.	Resentful	1	2	3	4	5
2.	Discouraged	1	2	3	4	5
3.	Shaky	1	2	3	4	5
4.	Worn Out	1	2	3	4	5
5.	Inadequate	1	2	3	4	5
6.	Excited	1	2	3	4	5
7.	Rebellious	1	2	3	4	5
8.	Blue	1	2	3	4	5
9.	Jittery	1	2	3	4	5
10.	Sad	1	2	3	4	5
11.	Uneasy	1	2	3	4	5
12.	Irritated	1	2	3	4	5
13.	Jealous	1	2	3	4	5
14.	Worried	1	2	3	4	5
15.	Frustrated	1	2	3	4	5
16.	Lonely	1	2	3	4	5
17.	Furious	1	2	3	4	5
18.	On edge	1	2	3	4	5
19.	Confused	1	2	3	4	5
20.	Nervous	1	2	3	4	5
21.	Angry	1	2	3	4	5
22.	Guilty	1	2	3	4	5
23.	Bored	1	2	3	4	5
24.	Helpless	1	2	3	4	5
25.	Upset	1	2	3	4	5

EuroQol EQ-5D-5L

Please click the ONE box that best describes your health TODAY.

MOBILITY

Choose one of these items / levels

I have no problems in walking about €
I have slight problems in walking about €
I have moderate problems in walking about €
I have severe problems in walking about €
I am unable to walk about €

SELF-CARE

Choose one of these items / levels

I have no problems washing or dressing myself €
I have slight problems washing or dressing myself €
I have moderate problems washing or dressing myself €
I have severe problems washing or dressing myself €
I am unable to wash or dress myself €

USUAL ACTIVITIES *(e.g. work, study, housework, family or leisure activities)*

Choose one of these items / levels

I have no problems doing my usual activities €
I have slight problems doing my usual activities €
I have moderate problems doing my usual activities €
I have severe problems doing my usual activities €
I am unable to do my usual activities €

PAIN / DISCOMFORT

Choose one of these items / levels

I have no pain or discomfort €
I have slight pain or discomfort €
I have moderate pain or discomfort €
I have severe pain or discomfort €
I have extreme pain or discomfort €

ANXIETY / DEPRESSION

Choose one of these items / levels

I am not anxious or depressed	€
I am slightly anxious or depressed	€
I am moderately anxious or depressed	€
I am severely anxious or depressed	€
I am extremely anxious or depressed	€

The best health you can imagine

100

90

80

70

60

50

40

30

20

10

0

The worst health you can imagine

- We would like to know how good or bad your health is TODAY.
- This scale is numbered from 0 to 100.
- 100 means the <u>best</u> health you can imagine.
  0 means the <u>worst</u> health you can imagine.
- Please click on the scale to indicate how your health is TODAY.
