# Supplementary material for: Efficacy of Telephone-Based Cognitive Behavioral Therapy for Weight Loss, Disordered Eating, and Psychological Distress After Bariatric Surgery: A Randomized Clinical Trial
Source: JAMA Netw Open. 2023 Aug 3;6(8):e2327099. doi: 10.1001/jamanetworkopen.2023.27099 (PMC10401302; doi:10.1001/jamanetworkopen.2023.27099)
Supplement: Supplement 2. — eTable 1. Raw Means for Clinical Variables eFigure. Estimated Mean Outcomes From a Linear Mixed Model Comparing Emotional Eating Scale Total Score [file jamanetwopen-e2327099-s002.pdf]

Supplemental Online Content

Sockalingam S, Leung SE, Ma C, et al. Efficacy of telephone-based cognitive behavioral therapy for weight loss, disordered eating, and psychological distress after bariatric surgery: a randomized clinical trial. *JAMA Netw Open*. 2023;6(8):e2327099. doi:10.1001/jamanetworkopen.2023.27099

eTable 1. Raw Means for Clinical Variables
eFigure. Estimated Mean Outcomes From a Linear Mixed Model Comparing Emotional Eating Scale Total Score

This supplemental material has been provided by the authors to give readers additional information about their work.

© 2023 Sockalingam S et al. *JAMA Network Open.*

eTable. Raw means ± standard deviations for clinical variables between Tele-CBT and Control groups at baseline, post-intervention, and 3-month follow-up.

**Measure**	**Baseline**		**Post-Intervention**		**Follow-Up**	
	Tele-CBT Mean (SD)	Control Mean (SD)	Tele-CBT Mean (SD)	Control Mean (SD)	Tele-CBT Mean (SD)	Control Mean (SD)
**%TWL**	N/A	N/A	1.44 (4.13)	1.11 (3.56)	1.01 (6.21)	0.95 (5.54)
**BES**	13.31 (8.70)	13.45 (8.64)	8.03 (6.21)	12.83 (9.53)	9.43 (7.52)	12.42 (9.52)
**EES-Total**	49.93 (18.39)	50.99 (22.40)	40.23 (15.72)	49.20 (22.99)	41.98 (15.58)	49.94 (22.23)
**PHQ-9**	5.73 (4.86)	5.94 (4.77)	3.60 (3.95)	6.24 (5.33)	4.51 (4.71)	6.79 (5.69)
**GAD-7**	4.75 (3.93)	4.78 (4.20)	2.94 (3.14)	5.37 (5.35)	3.40 (3.70)	5.40 (5.24)

%TWL: post-operative percent total weight loss; BES: Binge Eating Scale; EES-Total: Emotional Eating Scale total score; PHQ-9: Patient Health Questionnaire 9-item scale; GAD-7: Generalized Anxiety Disorders 7-item scale.

© 2023 Sockalingam S et al. *JAMA Network Open.*

eFigure. Estimated mean outcomes (± standard errors) from a linear mixed model comparing EES-Total (Emotional Eating Scale total score) between the Tele-CBT and Control groups over time. Timepoints 1, 2, and 3 refer to Baseline (1-year post-surgery), post-intervention (~15-months post-surgery), and 3-month follow-up (~18-months post-surgery), respectively. *p*-values refer to the group by time interaction.

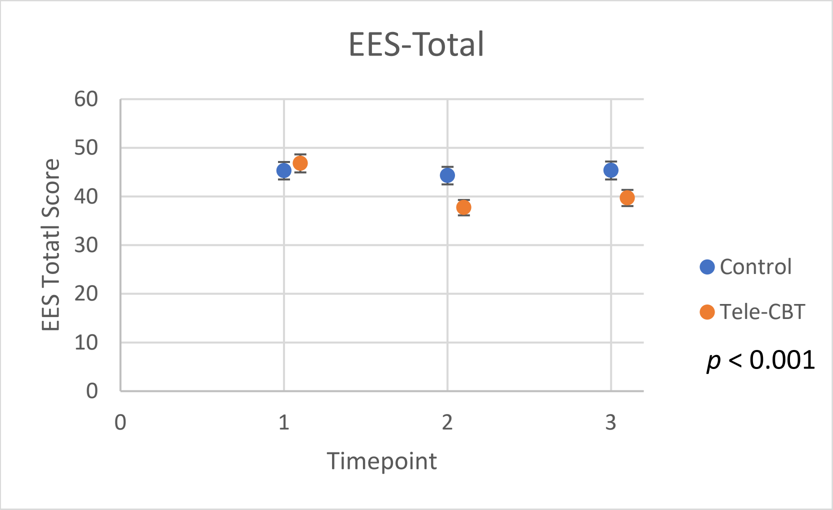

© 2023 Sockalingam S et al. *JAMA Network Open.*
