# Supplementary material for: Efficacy of Telephone-Based Cognitive Behavioral Therapy for Weight Loss, Disordered Eating, and Psychological Distress After Bariatric Surgery: A Randomized Clinical Trial
Source: JAMA Netw Open. 2023 Aug 3;6(8):e2327099. doi: 10.1001/jamanetworkopen.2023.27099 (PMC10401302; doi:10.1001/jamanetworkopen.2023.27099)
Supplement: Supplement 3. — Data Sharing Statement [file jamanetwopen-e2327099-s003.pdf]

Data Sharing Statement

Sockalingam. Efficacy of Telephone-Based Cognitive Behavioral Therapy for Weight Loss, Disordered Eating, and Psychological Distress After Bariatric Surgery: A Randomized Clinical Trial. *JAMA Netw Open*. Published online August 3, 2023 . doi:10.1001/jamanetworkopen.2023.27099

Data
Data available: No
